# Antibody-based delivery of interleukin-2 modulates the immunosuppressive tumor microenvironment and achieves cure in pancreatic ductal adenocarcinoma syngeneic mice

**DOI:** 10.1186/s13046-024-03238-x

**Published:** 2025-01-07

**Authors:** Carmine Carbone, Roberto De Luca, Emanuele Puca, Antonio Agostini, Alessia Caggiano, Lorenzo Priori, Annachiara Esposito, Serena Ascrizzi, Geny Piro, Lisa Salvatore, Francesco De Sanctis, Stefano Ugel, Vincenzo Corbo, Dario Neri, Giampaolo Tortora

**Affiliations:** 1 Department of Medical and Surgical Sciences, Medical Oncology , Fondazione Policlinico Universitario “Agostino Gemelli” IRCCS, Rome, Italy; 2 Philochem AG, Libernstrasse 3, Otelfingen, 8112 Switzerland; 3 Bioinformatics Research Core Facility, Gemelli Science and Technology Park (GSTeP), Fondazione Policlinico Universitario “Agostino Gemelli” IRCCS, Rome, Italy; 4 Department of Translational Medicine, Medical Oncology , Catholic University of the Sacred Heart, Rome, Italy; 5 Department of Medicine, Section of Immunology, University of Verona, Verona, Italy; 6 Department of Diagnostics and Public Health, University of Verona, Verona, Italy; 7 ARC-Net Research Centre, University of Verona, Verona, Italy; 8 Department of Chemistry and Applied Biosciences, Swiss Federal Institute of Technology, Zurich, CH-8093 Switzerland; 9 Philogen Spa, Piazza La Lizza 7, Siena, 53100 Italy

**Keywords:** Pancreatic cancer, Immunocytokines, Orthotopic syngeneic mouse models, Chemotherapy

## Abstract

**Background:**

Pancreatic ductal adenocarcinoma (PDAC) is one of the most aggressive and deadly type of cancer, with an extremely low five-year overall survival rate. To date, current treatment options primarily involve various chemotherapies, which often prove ineffective and are associated with substantial toxicity. Furthermore, immunotherapies utilizing checkpoint inhibitors have shown limited efficacy in this context, highlighting an urgent need for novel therapeutic strategies. This study investigates the preclinical efficacy of an innovative targeted therapy based on antibody-cytokine fusion proteins, specifically interleukin-2 (IL-2), a pivotal driver of cell-mediated immunity, fused to L19 antibody, which selectively binds to extra domain B of fibronectin (EDB-FN1) expressed in the tumor microenvironment.

**Methods:**

We tested the effectiveness of different immunocytokines through in vivo characterization in syngeneic C57BL/6J orthotopic mouse models of PDAC. Based on these results, we decided to focus on L19-IL2. To assess the efficacy of this immunocytokine we developed an *ex-vivo* immune-spheroid interaction platform derived from murine 3D pancreatic cultures, and telomerase reverse transcriptase (TERT) specific T-lymphocytes. Moreover, we evaluated the anti-cancer effect of L19-IL2 in combination with standard therapy in vivo experiments in PDAC mouse models. Tumor samples collected after the treatments were characterized for tumor infiltrating immune cell components by bulk RNA sequencing (RNA-seq) and spatial transcriptomics (Stereo-seq) analysis.

**Results:**

The tumor-targeted L19-IL2 fusion protein demonstrated potent, dose-dependent anti-tumor activity in mice with pancreatic tumors resistant to standard chemotherapy. Spatial Transcriptomics (ST) and RNA-seq analyses indicated that L19-IL2 treatment induced a significant influx of immune cells into the tumor microenvironment, with these cells expressing activation markers like granzymes, perforins, and the IL-2 receptors.

**Conclusions:**

Our results demonstrated that L19-IL2 enhances immune infiltration and cytotoxicity, remodeling the “cold” tumor microenvironment (TME) in PDAC. This innovative antibody-cytokine fusion protein improves therapeutic outcomes, paving the way for novel targeted treatment strategies in PDAC.

**Supplementary Information:**

The online version contains supplementary material available at 10.1186/s13046-024-03238-x.

## Introduction

PDAC is one of the most aggressive and deadly forms of cancer, accounting for over 85% of all solid pancreatic tumors [1, 2]. The prognosis for PDAC patients remains grim, with a five-year overall survival rate of just 11.5% for stages I-III and only 3.1% for those with metastatic stage IV disease [2]. Surgical resection is the only potentially curative treatment for PDAC, but unfortunately, only 20% of patients are candidates for surgery at the time of diagnosis [3].

First-line chemotherapy for PDAC typically involves either the FOLFIRINOX regimen, which combines 5-fluorouracil (5-FU), leucovorin, irinotecan, and oxaliplatin, or a combination of gemcitabine and nab-paclitaxel [4, 5]. When the disease progresses, patients receive second-line chemotherapy, though the optimal regimen has yet to be fully established. Second-line treatments vary by country, with 5-FU-based therapies commonly being used if these agents were not part of the first-line treatment [6]. Regimens such as FOLFOX (5-FU, oxaliplatin, and leucovorin) or 5-FU combined with liposomal irinotecan (Nal-IRI) are frequently employed [7, 8]. A recent meta-analysis showed that first -line PDAC patients treated with FOLFIRINOX or NALIRIFOX had a similar Progression Free Survival and Overall Survival [9].

Despite surgery and chemotherapy being the standard of care, the clinical benefits for PDAC patients remain very limited. Even novel immune checkpoint inhibitors that are clinically effective against various solid tumors exhibit limited efficacy in PDAC. This resistance is primarily due to a pronounced desmoplastic reaction and the formation of an immunosuppressive TME [10]. TME is rich in components of the extracellular matrix (ECM), such as fibronectin, which favor tumor survival and create barriers inhibiting immune cell recognition. Fibronectin is a key structural glycoprotein of ECM and plays several significant roles in the formation of this hostile environment [11]. These challenges underscore the urgent need for more effective therapeutic strategies.

Recombinant human IL2 (Proleukin®) was the first cytokine approved for the treatment of metastatic melanoma and renal cell carcinoma [12]. However, the therapeutic efficacy of recombinant cytokines is often limited by their toxicity already at low doses, which prevents escalation to therapeutically effective levels. Antibody-cytokine fusion proteins, known as immunocytokines, offer a potential solution by promoting selective localization to tumor sites, thereby minimizing damage to healthy tissues [13–19].

The alternatively spliced extra-domain B of fibronectin (EDB-FN1), whose 91-amino acid sequence is fully conserved between mice and humans, is a well-characterized marker of malignancy [20–22]. EDB-FN1 is undetectable in normal adult tissues but is strongly expressed in most aggressive solid and liquid tumors [23–25]. The fully human monoclonal antibody L19 [26], which targets EDB-FN1, has been shown to preferentially localize to tumor in both animal models and cancer patients [20, 27–29].

An immunocytokine based on the L19 antibody fused to IL-2 (L19-IL2), in combination with L19-TNF, has recently met the primary endpoint in a Phase III trial in melanoma [NCT03567889]. A second Phase III trial is ongoing in the United States in the same patient population [NCT02938299] along with two Phase II studies in non-melanoma skin cancers [NCT05329792, NCT04362722]. L19-IL2 has also been explored with gemcitabine in PDAC, but such combination did not yield objective responses [NCT01198522].

In this article, we have investigated various doses of L19-IL2, both as a monotherapy and in combination with FOLFOX, in syngeneic orthotopic mouse models of PDAC. L19-IL2 selectively localized to the neoplastic mass and demonstrated a dose-dependent inhibition of tumor growth. Furthermore, it transformed immunologically "cold" tumors into "hot" ones, as evidenced by immunofluorescence (IF), RNA-seq, and ST analyses. In summary, the findings of this study pave the way for new clinical investigations of L19-IL2 in patients with PDAC.

## Methods

### L19-IL2

L19-IL2 was kindly provided by Philogen S.p.A. The biochemical characterization of the product was conducted as previously described [30].

### PDAC mouse cell lines and 3D cultures

The murine PDAC cell lines DT4313, RC416, FC1242 and FC1245 were characterized and cultured according to Agostini et al. [31, 32]. Briefly, cancer cells were isolated from explants derived from KPC (LSL-Kras^G12D/+^; LSL-Trp53^R172H/+^; PDX1-CRE) and KC (LSL-Kras^G12D/+^; PDX1-CRE) mouse models. Both models utilize PDX1, a pancreatic-specific transcription factor that regulates multiple pancreatic cell lineages, including ducts, acini, and endocrine cells. The expression of PDX1 promotes the oncogenic mutations across different pancreatic cell types, resulting in a tumor with marked heterogeneity. This diversity within the tumor reflects the complex cellular composition of human PDAC, contributing to tumor aggressiveness and therapeutic resistance. The explants were initially cultured under two-dimensional (2D) conditions, and following a limited number of passages (maximum of five), distinct three-dimensional (3D) spheroids were subsequently generated. These 3D spheroids were directly derived from the initial 2D cultures, with cells first maintained in 2D before being transitioned to conditions that facilitate the development of 3D structures. The resulting 3D spheroids included the following lines: 13KC (DT4313), KPC416 (RC416), KPC06 (FC1242), and KPC12 (FC1245). The names in parentheses correspond to the specific cell lines established from the explants, which were then utilized to generate the respective 3D spheroids. Briefly, 2D cell lines were detached using trypsinization and suspended in 50 μl of Cultrex UltiMatrix Reduced Growth Factor Basement Membrane Extract (R&D systems Cat# BME001-10) at a density of 100,000 cells per dome. The spheroids were then grown in PancreaCult™ Organoid Growth Mouse Medium (STEMCELL Technologies, Cat#06040) and inspected daily. Fresh medium was added three times per week, and the spheroids were expanded only after complex structures had been established (10 to 14 days).

### RNA isolation and quantitative RT-PCR assay

Total RNA was extracted using the mirVana miRNA Isolation Kit (Thermo Fisher Scientific, Cat#AM1560-4), and cDNA was synthesized using the High-Capacity cDNA Reverse Transcription Kit (Thermo Fisher Scientific, Cat#4,368,814) according to the manufacturer’s instructions. Real-time quantitative PCR (qPCR) was performed on a QuantStudio3 system (Thermo Fisher Scientific, Cat#A28567) with specific primers and the Fast SYBR™ Green Master Mix (Thermo Fisher Scientific, Cat#4,385,610). Gene expression was calculated using the 2^−ΔΔCT^ method and normalized to β-actin expression.

The primers used were obtained using Primer-BLAST (RRID:SCR_003095) based on NCBI Reference Sequence: NM_010233.2:

- EDB_Isoform_FW (5′-TTGTCCCAGAGGTGCCCCAG-3′);
- EDB_Isoform_REV (5′-TCCCTTCTCCTGCCGCAACT-3′);
- EDB_Exclusion_FW (5′-TCCTGGCCTGGAGTACAACGT-3′);
- EDB_Exclusion_REV (5′-CGTGGGAGGAGGGACAGCTG-3′);
- EDA_Isoform_FW (5′-AGGCCGGGGTCTGAGTACAC-3′);
- EDA_Isoform_REV (5′-TGGGCGCAGGAATGGCTG-3′);
- EDA_Exclusion_FW (5′-CAGTGACCCCATTCCTGCGC-3′);
- EDA_Exclusion_REV (5′-TGGTCCTGTCTTCTCTTTCGGGT-3′).

The EDB_Exclusion primers were designed spanning the junctions of exon 25 (included in EDB-FN1). Similarly, EDA_Exclusion primers were designed to span the junctions of exon 33 (which is included in EDA-FN1). The inclusion ratio was determined by dividing EDB-FN1 reads by the total reads of all fibronectin isoforms.

### Immunohistochemistry (IHC) and Immunofluorescence analyses (IF)

For IHC and IF analysis a Formalin-fixed paraffin-embedded (FFPE) preparation of the spheroids was obtained performing a protocol described by Agostini et al. [31]. Whole 5 µm sections of the FFPE were dewaxed and rehydrated. Citrate Plus (10X) HIER Solution (ScyTek Laboratories, Cat#CPL500) was used for antigen retrieval, and 0.1% IGEPAL in PBS was used for permeabilization. The sections obtained were subjected to IHC and IF staining. The following antibodies were used for IHC staining with established procedures: Fibronectin (Abcam, Cat#ab268020, RRID:AB_2941028). For IF analysis on 3D cultures the following antibodies with established procedures were assessed: L19 (1:250). Images were acquired with EVOS FL Auto 2 Cell Imaging System (ThermoFisher Scientific, Cat#AMAFD2000). 20X GFP fluorescence raw images were quantified with QuPath (RRID:SCR_018257).

We performed IF analysis on tumor tissues excised from mice orthotopically injected with KPC06 and KPC12 following the procedures described before. The following antibodies were used for IF staining with established procedures: CD8 (Abcam, Cat#ab217344, RRID:AB_2890649), Perforin 1 (PRF1) (Abcam, Cat#ab16074, RRID:AB_302236) and Granzyme B (GRZB) (Cell Signaling Technology, Cat#17,215, RRID:AB_2798780). Images were acquired with EVOS FL Auto 2 Cell Imaging System (Thermo Fisher Scientific, Cat#AMAFD2000) and processed with QuPath (RRID:SCR_018257) for cell segmentation and positive cell count.

IF analysis on freshly frozen sections of tumor tissues derived from patients and mouse models for EDB-FN1 expression and IF-based biodistribution analysis on KPC06 mouse model were performed as previously described [33].

### Ex-vivo immunity-spheroid interaction platforms

#### Mouse telomerase reverse transcriptase (TERT) specific T-lymphocytes establishment and culture

Polyclonal mTERT_198–205_-specific CTLs were expanded from C57BL/6J vaccinated-splenocytes by mixed-leukocyte peptide culture in the presence of 0.1 μM of mTERT198-205 peptide (VGRNFTNL) (JPT, Peptide Technologies) according to De Sanctis et al. [34]. Cells were kept in weekly expansion by co-culture of CTLs with irradiated, syngeneic splenocytes pulsed with 0.1 μM TERT peptide in complete medium containing 20 IU/ml of recombinant human IL-2 (Miltenyi Biotec, Cat#130–097–743). OVA_257–264_-specific CTLs derived from OT-1 splenocytes were stimulated once with 1 μM specific OVA peptide (SIINFEKL) (JPT, Peptide Technologies) in complete medium containing 20 IU/ml of recombinant human IL-2 (Miltenyi Biotec, Cat#130–097–748)) and were used as control (CTR).

#### Spheroids/T-lymphocytes interaction platform establishment

Murine 3D cultures were plated in the Xeno-free matrix Vitrogel ORGANOID-3 (The WellBioScience, Cat#VHM03) at a concentration of 500 spheroids/plate in 48well plate, according to manufacturer’s instructions. The spheroids were treated with L19-IL2 at a final concentration of C = 50 µg/ml for 2 h at 37 °C. Activated T-lymphocytes were labeled with 500 nM CellTracker™ Red CMPTX Dye (Invitrogen, Cat#C34552) according to manufacturer’s instruction, and then added to 3D cultures (Effector:target ratio 500:1). Apoptosis was detected using CellEvent™ Caspase-3/7 Green ReadyProbes™ Reagent (Invitrogen, Cat#R37111) and fluorescence images were acquired using the EVOS FL Auto 2 Cell Imaging System (Thermo Fisher Scientific, Cat#AMAFD2000) for 48 h post-interaction. 20X GFP fluorescence raw images were processed using QuPath(RRID:SCR_018257) for cell segmentation and positive cell count.

### In vivo experiments

For the generation of orthotopic syngeneic models we used C57BL/6J mice (RRID:IMSR_JAX:0006649) following the procedure described by Agostini et al. [31]. Mice were euthanized at the indicated time points. After 7 days following transplantation, tumor-bearing mice were subjected to high-contrast ultrasound imaging using the Vevo 3100 LT Imaging System (VisualSonics) and randomly assigned to the experimental group in order to correct the dimension bias. Treatment was initiated when tumor volume reached ~ 50mm^3^, which represents the inclusion criterion cut-off together with the compliance with human endpoint according to ethical animal guidelines.

All the animal models used for the experiments met the inclusion criterion with no loss of data point. In order to characterize different immunocytokines sensitivity, KPC06 mice were randomly assigned by GraphPad random number generator (https://www.graphpad.com/quickcalcs/randomize1/) to receive once a week for 2 weeks: vehicle (CTR), L19-IL2 (100 µg/mouse), L19mIL12 (12 µg/mouse), mIL2-F8-mTNF(mut) (40 µg/mouse), L19mTNF (4 µg/mouse), standard chemotherapy with gemcitabine 10 mg/kg + abraxane 3 mg/kg (Gem/Abx).

Upon selecting the appropriate dose for each PDAC model, a low dose for KPC06 (30 µg/mouse) and a high dose for KPC12 (100 µg/mouse), mice were treated according to each group (*n* = 8 mice each group): control group (Vehicle), standard therapy (FOLFOX i.p., once a week for 2 weeks), L19-IL2 (30/100 µg/mouse i.v., once a week for 2 weeks) and combination group (Co-delivery of L19-IL12 and FOLFOX subsequentially, once a week for 2 weeks). After 2 weeks of treatment, three mice per group were euthanized and biological materials collected for downstream analysis (RNA-seq, ST analysis, IF analysis). No significant body weight differences were detected on treatments. The primary outcome was survival duration. Tumor size was measured with Vevo 3100 LT Imaging System (VisualSonics) ultrasound device weekly in blind for group allocation and animals were sacrificed when tumor volume reached the prefixed cut-off volume. Weighting, measuring and treatment order were randomized each experimental session, with each animal tested at a different time each test day to minimize confounding factors. Same mice were housed together in individually ventilated cages with two or four mice per cage. All mice were maintained on a regular diurnal lighting cycle with ad libitum access to food and water. Environmental enrichment included nesting material. In vivo experiments were designed according to ARRIVE reporting guidelines [35].

### RNA-seq

Excised KPC06 tumor bulks (*n* = 3 mice for each condition) were collected after 2 weeks of treatments and stored in RNAlater Stabilization Solution (Thermo Fisher Scientific, Cat#AM7021). RNA was extracted with miRNeasy Micro Kit (Qiagen, Cat#217,084) to perform transcriptome sequencing (3’mRNA-Seq) with QuantSeq 3’mRNA-Seq V2 Library Prep Kit REV (Lexogen, Cat#225.24). Fastq files were processed and aligned with QuantSeq pipeline, and transcripts counts were imported in R with DESeq2 (RRID:SCR_015687) [36] to perform Differential Expression Analysis (DEA). Gene Set Enrichment Analysis (GSEA) was performed with the R package clusteRprofiler (RRID:SCR_016884) to get insight into the biological processes modulated by the different treatments. EDB-FN1 expression was calculated from total RNA-seq data using the bash command grep as described in Panagopoulos et al. [37] The reads spanning junctions of exon 25 (included in EDB-FN1) and the reads supporting the exclusion (junctions of exon 24–26) were identified. Inclusion ratio was calculated by dividing inclusion reads by the exclusion reads after normalization by the total reads covering Fn1 gene (RefSeq GeneID: 14,268).

### Spatial transcriptomics

Excised KPC06 tumors were collected one week after L19-IL2 therapy discontinuation and included in FFPE after fixation in 4% paraformaldehyde (PFA) for 14 h. Serial sections for each sample (*n* = 3 for each condition) were obtained to identify the region of interest with both tumor and TME representation. Four samples (*n* = 1 for each condition) were chosen to build a tissue macro array (TMA) in FFPE for ST analysis with Stereo-Seq OMNI (STOmics). TMA 5 μm section was positioned on Stereo-Seq Chip T (1cmx1cm) and sequencing library was prepared following the protocol at STOmics Riga laboratory. Fastq files were processed with Stereo-Seq Analysis Workflow (SAW) (RRID:SCR_025001) for alignment and barcode positioning. Processed files (gef) were analyzed with Stereopy v 1.3.1 to perform clustering and spot annotation. Briefly, 100 bin was chosen as optimal parameter for data analysis, low counts areas (necrotic area on L19-IL2 sample) were excluded from the analysis using the Stereopy cut function. Imported data counts were preprocessed with gaussian smoothing [38] and clustering was performed using Phenograph v 1.5.2 algorithm (RRID:SCR_016919). Cluster annotation was performed with SingleR (RRID:SCR_023120) using Azimuth mouse references.

### Statistical analysis

All results, when applicable, were expressed as the means ± Standard Deviation (SD). All statistical analyses and Kaplan–Meier curves were performed using GraphPad Prism (RRID:SCR_002798). *P*-values less than 0.05 were considered statistically significant. In particular, *P*-value < 0.05 was indicated in figures with one asterisk (*), *P*-value < 0.01 with two asterisks (**), *P*-value < 0.001 with three asterisks (***) and *P*-value < 0.0001 with four asterisks (****). A Student’s t-test or One-Way ANOVA test were applied to calculate the statistical significance between multiple group comparisons. Differences in survival duration were determined using Log-rank (Mantel-Cox) test using GraphPad Prism (RRID:SCR_002798). Mice sample size estimation was calculated by power analyses (G-power software) (RRID:SCR_013726) and based on our previous papers.

## Results

### Analysis of EDB-FN1 expression in spheroids models of pancreatic cancer

**Fig.1:**
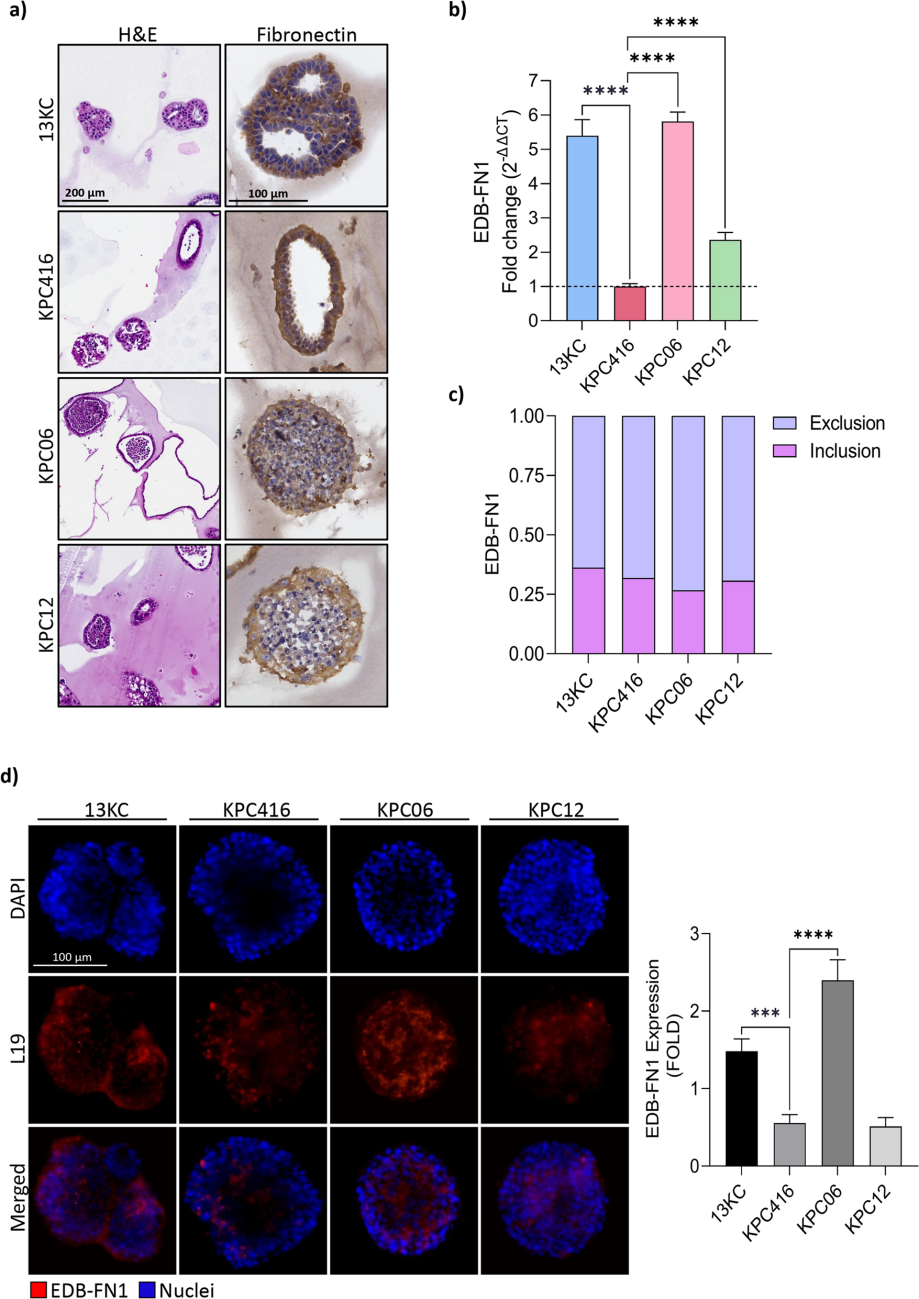
Characterization of the expression of EDB-FN1 in mouse pancreatic cancer models. **a** Histochemical analysis of Fibronectin expression in different 3D models of PDAC. 10X images of Hematoxylin/Eosin, 40X images of Fibronectin. Images shown are representative of 1 out of more than 10 fields acquired. **b** Real-time analysis of EDB-FN1 in spheroid models of PDAC. **c** Analysis of inclusion splice junction (pink bars) and exclusion splice junction (light blue bars) reads of EDB-FN1. **d** IF analysis on spheroids confirmed L19 was able to recognize EDB-FN1. Protein analyzed (in red) and nuclei (in blue) were reported. Images shown are representative of 1 out of more than 10 fields acquired. Bar plot showing the fold increase in EDB-FN1 fluorescence calculated as the ratio between the mean of CTCF quantified in each group and the mean of CTR. *P*-value < 0.05 was indicated in figures with one asterisk (*), *P*-value < 0.01 with two asterisks (**), *P*-value < 0.001 with three asterisks (***) and *P*-value < 0.0001 with four asterisks (****)

Initially, we validated the chemical features of the drug. Supplementary Fig. 1a shows the molecular structure of L19-IL2, a clinical-stage immunocytokine [39]. The non-covalent homodimer runs as a monomer in SDS-Page (Supplementary Fig. 1b) with a molecular size of ~ 42 kDa, while the SEC profile (Supplementary Fig. 1c) shows the dimeric product (~ 84 kDa). We confirmed the biological activity of L19-IL2 through a proliferation assay on CTLL2 cells (Supplementary Fig. 1d). The fusion protein exhibited IL-2 activity that closely matched the previously reported results for this product [30, 40, 41]. The 3D cultures better recapitulate the complexity of the TME and the interactions between cancer cells and stromal components [42]. First of all, we conducted IHC analysis to assess the expression of the stromal component, specifically of fibronectin, in spheroid models (13KC, KPC416, KPC06 and KPC12) (Fig. 1a). Afterwards, we evaluated the expression of EDB-FN1 using RT-PCR analysis (Fig. 1b) and determined the ratio of exclusion and inclusion of the alternative splicing isoform relative to the expression of total fibronectin (Fig. 1c). The results demonstrated that our models not only express EDB-FN1, but also exhibit an elevated isoform expression ratio compared to total fibronectin. Additionally, we performed IF analysis to assess the L19 ability to specifically recognize EDB-FN1 expressed in our spheroids (Fig. 1d). Among the available models, we selected KPC06 (Low immunogenic) and KPC12 (Non immunogenic) cancer cells, which closely mimic human pancreatic tumors. Indeed, these models do not elicit a robust immune response and, as previously shown, are resistant to immunotherapy [32, 43].These analyses collectively confirm that immunosuppressive KPC06 and KPC12 express EDB-FN1 and are suitable models for testing the L19-targeted antibody.

### Establishment of an ex-vivo immunity-spheroid interaction platform

To investigate L19-IL2 effects in a complex system that recapitulates the main tumor and immune cell components, we developed an immune-spheroid interaction platform with KPC06 and KPC12 and tumor-antigen cytotoxic T-lymphocytes (CTLs), as described in Agostini et al. [31]. Briefly, PDAC expresses several tumor-associated antigens (TAAs), and among them, TERT has been extensively studied for immunotherapy [40, 41]. In line with these premises, we used mouse TERT specific T-lymphocytes [42] cultured as described by De Sanctis et al. [43]. To evaluate the effect of L19-IL2 on the activity of T-lymphocytes in the recognition of cancer cells we treated KPC06 and KPC12 with L19-IL2 for 2 h. After the treatment, TERT specific T-lymphocytes [34] labelled with CellTracker Red CMPTX Dye were added to the platform. The spheroids were previously labeled with CellEvent Caspase-3/7 Green ReadyProbes and analyzed by time-lapse live microscopy to measure apoptosis induction.

**Fig. 2:**
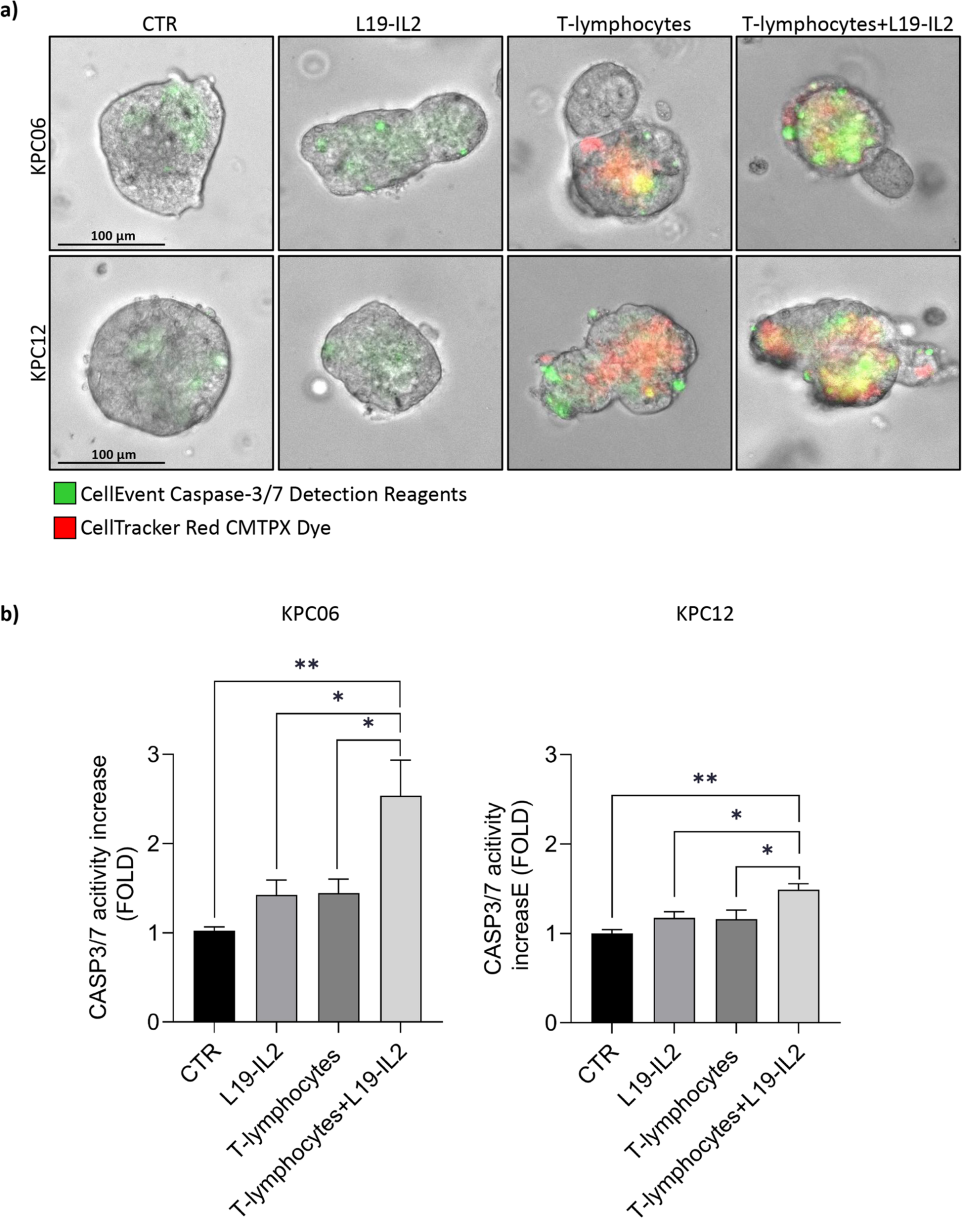
Evaluation of the L19-IL2 effect on *ex-vivo* interaction platforms. **a** Immunity-spheroid interaction platforms with TERT specific T-lymphocytes and KPC06 and KPC12 treated with L19-IL2. The induction of apoptosis was evaluated using the CellEvent Caspase-3/7 Detection Reagent (green), while T-lymphocytes were stained with the vital staining CellTracker Red CMPTX Dye (Red). The platforms were monitored daily, and fluorescence images were acquired using the EVOS FL Auto 2 Cell Imaging System over a 48 h period. Images shown are representative of 1 out of more than 10 fields acquired. **b** Bar plot showing the fold increase in Caspase 3/7 activity in comparison to CTR. The fold increase is calculated as the ratio between the mean of CTCF quantified in each group and the mean of CTR. *P*-value < 0.05 was indicated in figures with one asterisk (*), *P*-value < 0.01 with two asterisks (**), *P*-value < 0.001 with three asterisks (***) and *P*-value < 0.0001 with four asterisks (****)

As expected, T-lymphocytes were able to recognize and engage with the cancer cells, triggering a significant increase in apoptotic cell death. However, treatment with L19-IL2 resulted in a marked enhancement of the cytotoxic activity of T-lymphocytes. This augmented killing capacity led to a pronounced increase in apoptosis specifically in the KPC06 and KPC12 tumor cell lines (Fig. 2a).

The impact of L19-IL2 on T-lymphocytes recruitment was found to be consistent across the two 3D models assessed, KPC06 (Low immunogenic model) and KPC12 (Non immunogenic model) (Fig. 2b).

Overall, these findings suggest that L19-IL2 can increase T-lymphocytes infiltration and antitumor activity in 3D pancreatic tumor models.

### The L19-targeted antibody specifically hits EDB-FN1 of mouse and human cancer tissues

**Fig. 3:**
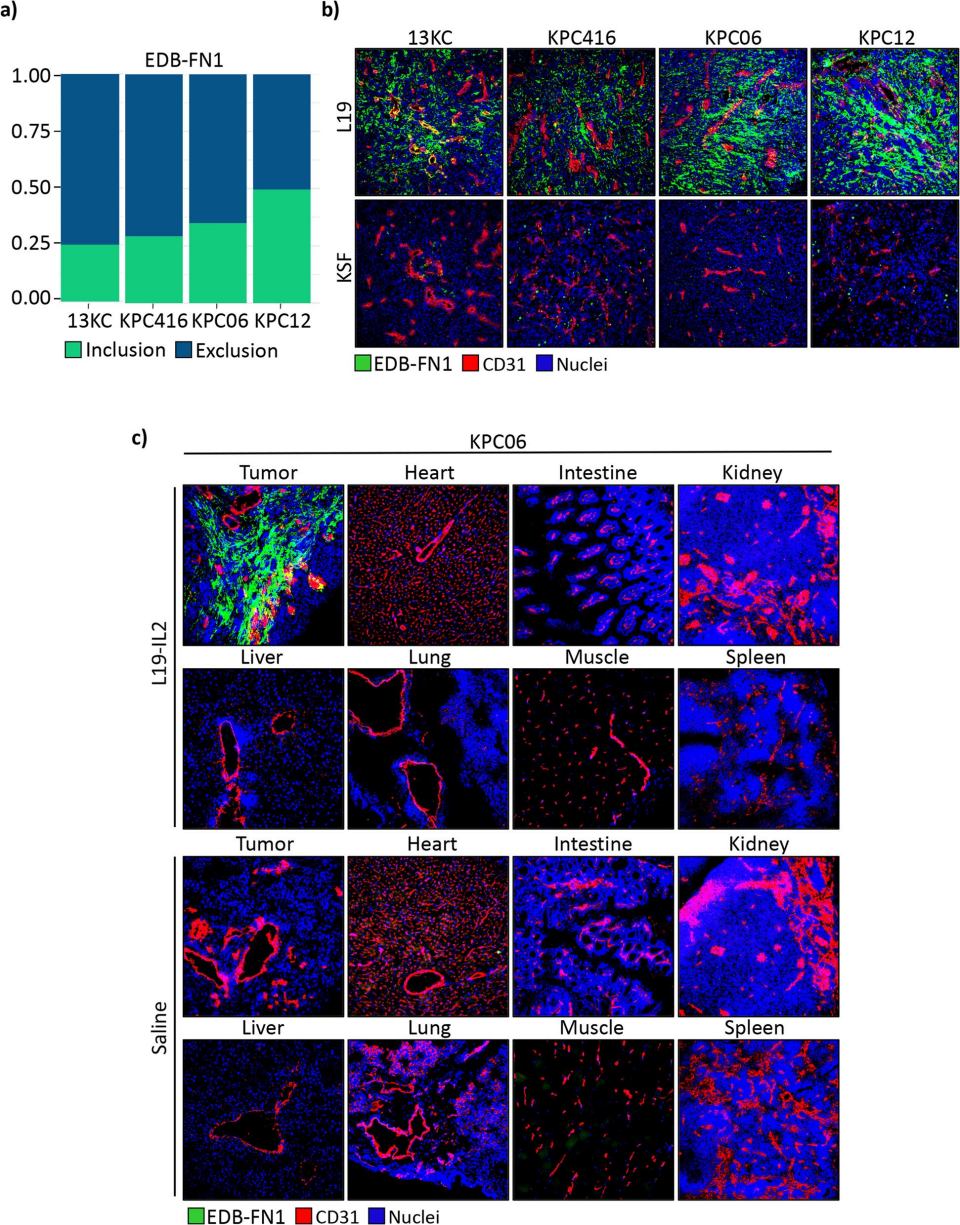
The L19-targeted antibody specifically hits EDB-FN1 of mouse cancer tissues. **a** Graphic representation of inclusion splice junction (green bars) and exclusion splice junction (blue bars) reads of EDB-FN1 from RNA-seq raw data in PDAC model tissues. **b** L19-targeted antibody in IgG1 format specifically target EDB-FN1 (Green) in vivo mice tumor tissues, while KSF antibody (specific for hen egg lysozyme, an irrelevant antigen) was used as negative CTR. **c** IF-based biodistribution analysis in orthotopic KPC06 mice. EDB-FN1 shown in green, saline was used as negative CTR

We assessed the expression of EDB-FN1 in tumor tissues derived from our PDAC models by both RNA-seq (Fig. 3a) and IF (Fig. 3b) using the L19 antibody, while the KSF antibody (specific to hen-egg lysozyme) was used as negative control. Moreover, we assessed the EDB-FN1 expression pattern also in PDAC patient samples (Supplementary Fig. 2).

We performed an IF-based biodistribution analysis in mice bearing KPC06 tumors. L19-IL2 showed a preferential accumulation in tumors 24 h after intravenous administration. No uptake could be detected in healthy organs or in animals injected with saline solution (Fig. 3c).

These results suggest that L19-IL2 has the potential to be an effective targeted treatment for pancreatic tumors, with high selectivity for tumor cells over normal tissues.

### In vivo characterization of immunocytokines sensitivity in syngeneic orthotopic mouse PDAC models

To test the effectiveness of different immunocytokines in pancreatic tumor models, C57BL/6J mice (RRID:IMSR_JAX:000664) were orthotopically injected with KPC06 and randomly assigned to receive once a week for 2 weeks: vehicle, as CTR, L19-IL2 high dose (100 µg/mouse), L19mIL12 (12 µg/mouse), mIL2-F8-mTNF(mut) (40 µg/mouse), L19mTNF (4 µg/mouse), standard chemotherapy with gemcitabine 10 mg/kg + abraxane 3 mg/kg (Gem/Abx) (Supplementary Fig. 3).

As expected, standard chemotherapy did not prove effective in reducing tumor volume when compared to the CTR group. As for the different immunocytokines, except for L19mTNF which failed to lead to positive results, the others (L19-IL2, L19mIL12, and mIL2-F8-mTNF(mut)) demonstrated almost complete tumor elimination (Supplementary Fig. 3a).

Additionally, the impact of these immunocytokines on the median survival rate was evaluated (Supplementary Fig. 3b). We observed that standard therapy (Gem/Abx) and L19mTNF failed to prolong mice median survival rate. On the contrary, L19-IL2, L19mIL12, and mIL2-F8-mTNF(mut) were able to cure all tumor-bearing mice. They were ultimately sacrificed when tumor volume reached the cut-off. To assess the possible adverse effects of the different immunocytokines, we measured changes in body weight, thus finding no substantial weight loss (Supplementary Fig. 3c). In summary we tested various immunocytokines in pancreatic tumor models, finding that, unlike standard chemotherapy, L19-IL2, L19mIL12, and mIL2-F8-mTNF(mut) effectively eliminated tumors and extended survival, while L19mTNF and standard therapy did not improve outcomes.

### Dose-dependent reduction of tumor volume in syngeneic orthotopic mouse PDAC models following L19-IL2 treatment

Based on the previous results, we chose to focus on L19-IL2 for several reasons. PDAC are notoriously resistant to standard therapies and often display poor T-lymphocyte activation and limited recruitment of natural killer (NK) cells. Therefore, the ability to selectively target EDB-FN1 within the TME and locally increase IL-2 concentrations—thereby enhancing the activation and proliferation of effector cells—could be a key strategy for effective tumor eradication. The importance to selectively deliver IL-2 to the site of disease using the L19 antibody has been previously demonstrated in multiple murine tumor models [39, 44–46]. Moreover, based on the promising results obtained with the high dose of L19-IL2 (100 µg/mouse) in KPC06 mice, we decided to test a lower dose (30 µg/mouse) to evaluate its impact on tumor volume reduction. Our findings revealed that L19-IL2 low-dose resulted in a smaller, yet still significant reduction in tumor volume growth when compared to L19-IL2 high-dose (Supplementary Fig. 4).

### L19-IL2 potentiates the activity of FOLFOX in syngeneic orthotopic mouse PDAC models

To evaluate the effects of L19-IL2 in combination with standard therapy (FOLFOX) on tumor volume and survival rates of PDAC models with different immunogenic potential, C57BL/6J mice (RRID:IMSR_JAX:0006649) were orthotopically injected with KPC06 (Low immunogenic model) and KPC12 (Non immunogenic model). In order to assess the effects of the combination, different dosage levels of the immunocytokine were selected: L19-IL2 low dose (30 µg/mouse) for KPC06 and L19-IL2 high dose (100 µg/mouse) for KPC12.

We selected a low dose for KPC06 based on the results from the dose–response experiment, while a high dose was chosen for KPC12 due to its characteristics as a non-immunogenic and more aggressive model with lower EDB-FN1 expression.

**Fig.4:**
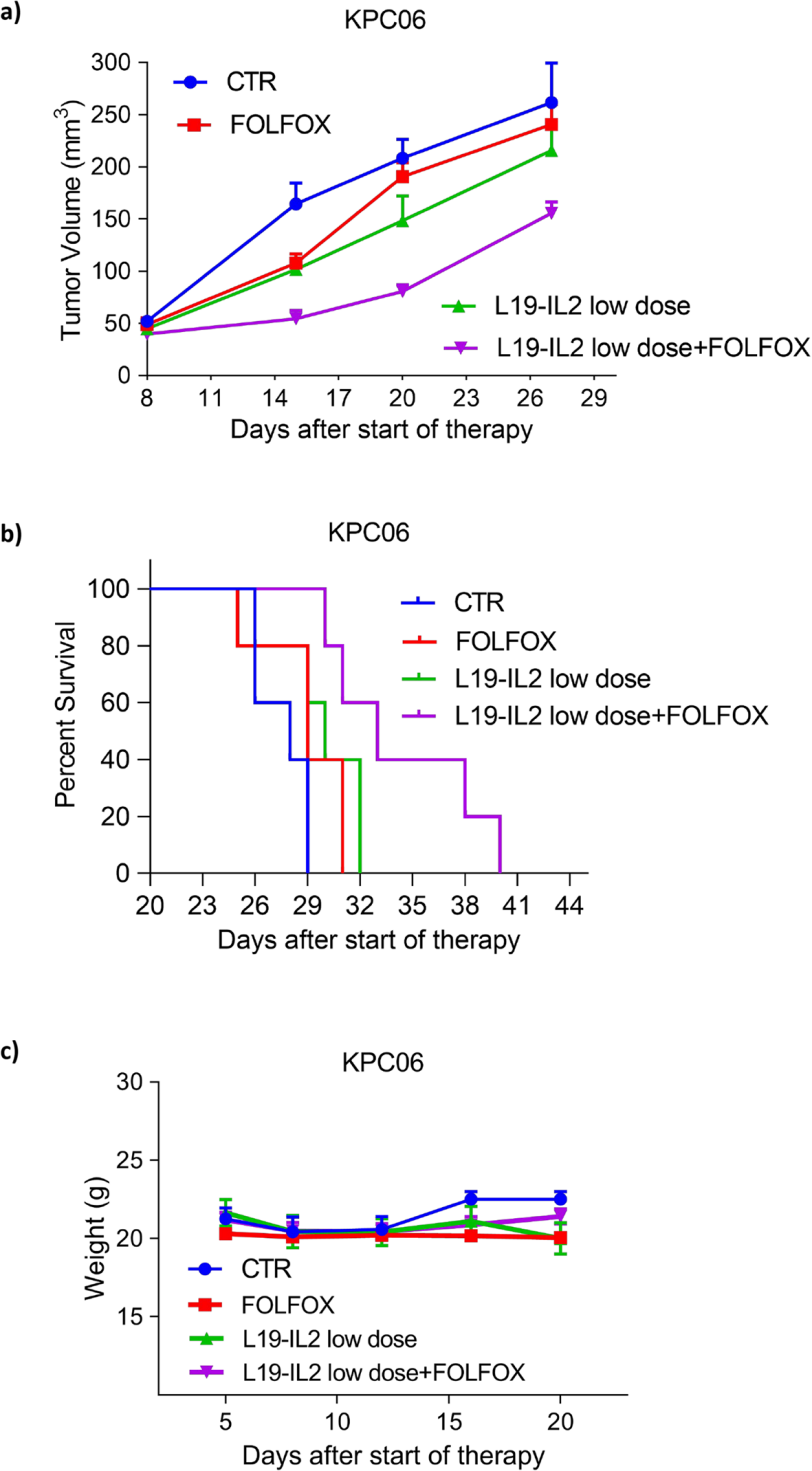
L19-IL2 treatment effects in combination with FOLFOX in KPC06 model. Plot showing tumor growth curves of KPC06 tumor-bearing mice randomly assigned to receive vehicle, as CTR, standard therapy (FOLFOX i.p., once a week for 2 weeks), and L19-IL2 (30 µg/mouse i.v., once a week for 2 weeks) alone or in combination with FOLFOX; **b** Kaplan–Meier survival analysis of KPC06 mice, grouped according to each experimental condition. **c** Variation of body weight in the different treatment groups

The mice were randomly divided into 4 groups (*n* = 8 mice in each group). The groups were treated with standard therapy (FOLFOX i.p., once a week for two weeks), or vehicle, as CTR, and L19-IL2 low dose for KPC06 and a high dose for KPC12 (i.v., 2 injections with 5-day interval) alone or in combination with FOLFOX. Treatments started when tumor volume reached ~ 50mm^3^. To evaluate the potential side effects of the treatment, body weight loss was assessed in both low and non immunogenic models (Fig. 4c and Supplementary Fig. 5c). No significant weight loss occurred in any treatment group, thus indicating the safety of the therapy.

L19-IL2 as a single agent showed a reduction in tumor growth that was comparable to that obtained with FOLFOX treatment. Additionally, when combined with FOLFOX, L19-IL2 led to a statistically significant decrease in tumor growth compared to the other treatments administered alone (Fig. 4a). The effect of L19-IL2, FOLFOX and combination therapy on median survival rate was also evaluated (Fig. 4b). Our findings revealed that FOLFOX (28 days vs 29 days; Chi square = 2.064; df = 1; *P*-value = 0.1508) and L19-IL2 treatment (28 days vs 30 days; Chi square = 3.493; df = 1; *P*-value = 0.0616), when used as single agents, were unable to extend mouse median survival. However, the combination treatment proved to be more effective than the individual treatments and significantly prolonged mouse median survival (28 days vs 33 days, Chi square = 9.151; df = 1; *P*-value = 0.0025).

Overall, here we demonstrated that L19-IL2 alone reduced tumor growth similarly to FOLFOX, but when combined with FOLFOX, it significantly decreased tumor growth and extended median survival compared to either treatment alone.

The KPC12 model presented no statistically significant reduction in tumor growth in any treatment group at day 18 (Supplementary Fig. 5a). Afterwards we were unable to verify the effects on tumor volume as the mice in the control and FOLFOX groups died before the others, so measurement data were not available.

We observed that L19-IL2 resulted in a significant increase in survival, both as a single agent (22 days vs 30 days, Chi square = 5.552; df = 1; *P*-value = 0.0185) and in combination (22 days vs 39 days, Chi square = 5.552; df = 1; *P*-value = 0.0185). While, as well as in the low immunogenic model, no significant differences were observed compared to FOLFOX (22 days vs 19 days, Chi square = 0.9724; df = 1; *P*-value = 0.3241) (Supplementary Fig. 5b).

### L19-IL2 increases immune infiltrate into the tumor core

**Fig. 5:**
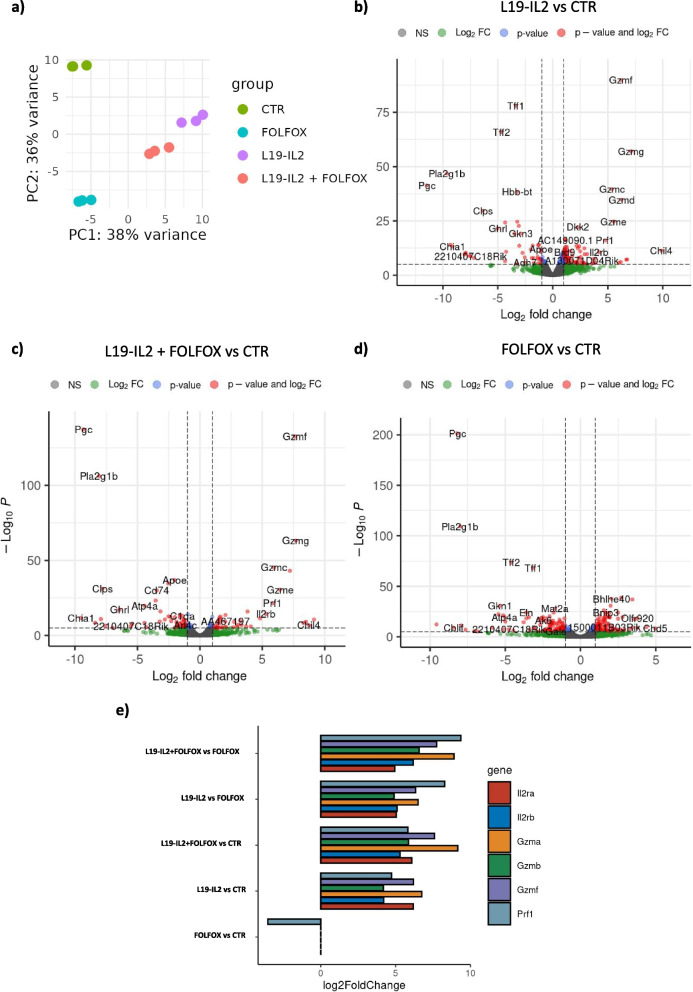
Differential expression analysis on KPC06 treated mice show immune response activation. **a** Plot showing Principal Component Analysis on RNA-seq data. **b** Volcano plot showing the genes differentially expressed (log2 Fold Change ≦ −1.5 ≧ 1.5, FDR < 0.05) in the comparison between L19-IL2 treated mice and control (CTR). **c** Volcano plot showing the genes differentially expressed (log2 Fold Change ≦ −1.5 ≧ 1.5, FDR < 0.05) between mice treated with a combination of L19-IL2 and FOLFOX and CTR. **d** Volcano plot showing the genes differentially expressed (log2 Fold Change ≦ −1.5 ≧ 1.5, FDR < 0.05) between FOLFOX treated mice and CTR. **e** Bar plot showing log2 Fold Change value for T-lymphocyte activation genes resulting from DEA between the different treatment groups

Tumor bulks (n = 3) from KPC06 mice treated with FOLFOX, L19-IL2, and the combination of both agents were characterized by 3’mRNA-seq to unravel the effects of L19-IL2 on pancreatic tumors (Fig. 5a). The analysis clearly showed that there was a consistent effect of L19-IL2 alone or in combination with FOLFOX on immune activation, and cytotoxic activity. A total of 117 and 101 genes were upregulated in L19-IL2 and L19-IL2 + FOLFOX treated mice respectively in contrast to CTRs (Fig. 5b and c). Among these we found a considerable over regulation of IL-2 receptors Il2Ra (CD25) and Il2Rb (CD122) and cytotoxic-related genes (Fig. 5e) highlighting the effective immune activating function of L19-IL2. On the contrary, among the 158 genes upregulated in FOLFOX mouse there was a decrease in cytotoxicity genes (Fig. 5d). In fact, a consistent increase of expression of immune response and T-lymphocyte activation signatures were found in the comparison between L19-IL2 and FOLFOX (Supplementary Fig. 6) and L19-IL2 plus FOLFOX and FOLFOX alone (Supplementary Fig. 7).

**Fig. 6:**
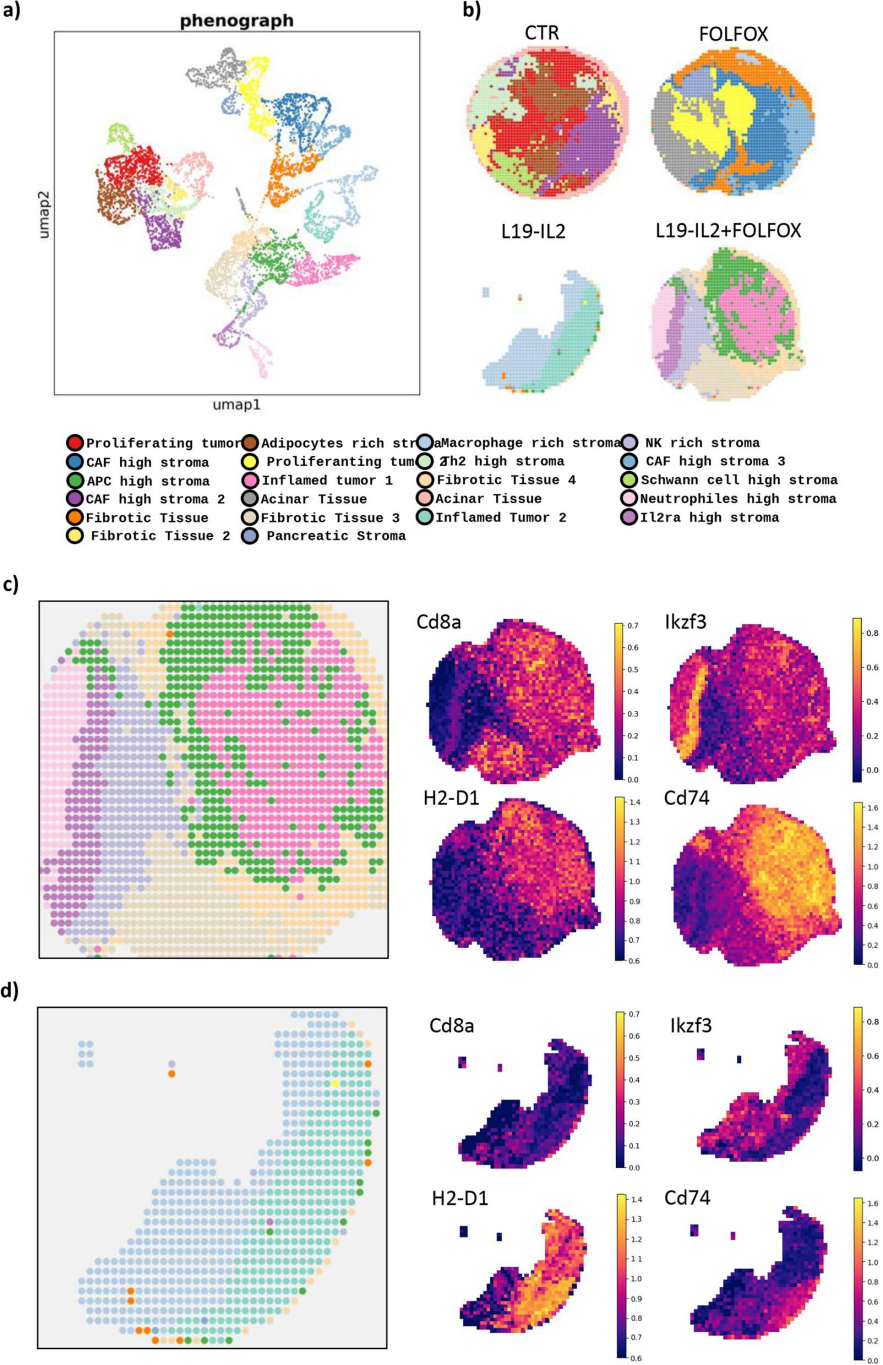
Stereo-Seq OMNI spatial transcriptomics analysis. **a**,**b** Spatial clustering of Stereo-seq OMNI data on the four cores analyzed. **c** Spatially resolved clusters of L19-IL2 + FOLFOX sample and heatmap showing an enhanced inflammation in the tumor core with a major representation of Cd8a^+^ Activated T-lymphocytes, Cd74^+^ H2-D1^+^ APCs and Ikzf3^+^ NK. **d** Spatially resolved clusters of L19-IL2 sample and heatmap showing an inflamed tumor core

To further validate these findings, we analyzed selected tumor tissue regions with Stereo-Seq OMNI ST technology. With this technology, we were able to characterize the cell population heterogeneity associated with the effect of L19-IL2 and FOLFOX with a resolution of 50 μm. We identified a total of 22 different clusters annotated according to the main cell type identified using SingleR (Fig. 6a and b). We found that L19-IL2 enhances immune infiltration in concomitance of FN1 expression, similarly to what we have found with bulk 3’mRNA-seq. The administration of L19-IL2 in combination with FOLFOX or as single agent had a potent effect on recruitment and activation of both CD8a^+^ T-lymphocytes and NK into the tumor front (Fig. 6c and d; Supplementary Fig. 8b-d), while those cells where not present in both CTR and FOLFOX treated tumors.

The presence of such cells localized with the high expression levels of both Il2Ra and Il2Rb (Supplementary Fig. 8a), two IL-2 receptors that orchestrate activation of both T-lymphocytes and NK, entailing the attractant and activating role of L19-IL2 role in tumors [39, 44, 45]. Moreover, ST showed how that L19-IL12 induced a consistent increase of Antigen-presenting cells (APCs) expressing MHC-II genes (Cd74, H2-Eb1, H2-Ab1), MHC-I genes (H2-D1, H2-K1) and co-stimulatory CD80 and CD86 potentiating T-lymphocytes activation signaling (Fig. 6c and d, Supplementary Fig. 8e).

**Fig. 7:**
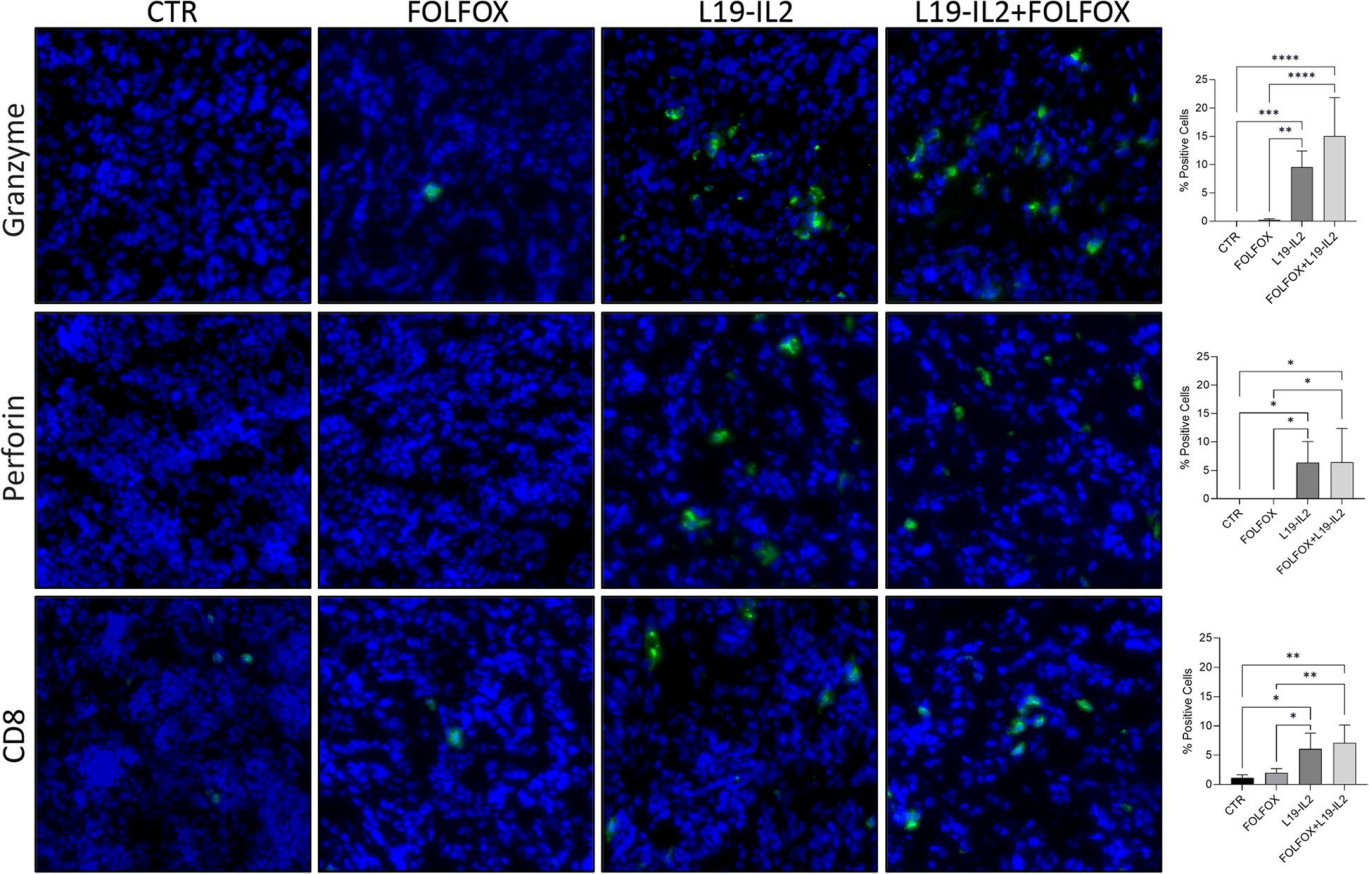
Immunofluorescence analysis on KPC06 models. IF analysis for CD8.^+^ TILs, GRZB and PRF1 in KPC06 tumor tissues. Protein analyzed (in green) and nuclei (in blue) are reported. Images shown are representative of 1 out of more than 10 fields acquired. Bar plots show percentage of positive cells grouped by treatments. *P*-value < 0.05 was indicated in figures with one asterisk (*), *P*-value < 0.01 with two asterisks (**), *P*-value < 0.001 with three asterisks (***) and *P*-value < 0.0001 with four asterisks (****)

By ST we demonstrated that KPC06 tumor bearing mice treated with L19-IL2 alone, or especially when combined with FOLFOX, significantly enhanced immune activation and cytotoxicity, evidenced by upregulation of key immune-related genes and increased infiltration of CD8a^+^ T-lymphocytes and NK cells into the tumors, while FOLFOX alone mainly boosted cytotoxicity genes. To validate these findings, we performed IF analysis on FFPE tumor samples of KPC06 (Fig. 7) and KPC12 models (Supplementary Fig. 9). Specifically, we aimed to verify the expression of CD8^+^ TILs, GRZB^+^ cytotoxic effector cells, and PRF1^+^ cytotoxic effector cells across the different treatment groups confirming the major effect on T-lymphocyte activation of L19-IL2 plus FOLFOX therapy.

We demonstrated that L19-IL2 affects tumor growth and immune cell infiltration in KPC06 tumors using RNA-seq, ST, and IF. L19-IL2 significantly increased immune cell infiltration, particularly CD8^+ ^T-lymphocytes and cytotoxic cells, and enhanced the efficacy of FOLFOX. The combination treatment showed the greatest increase in immune cells, while L19-IL2 alone had a smaller effect in the less immunogenic KPC12 model.

## Discussion

PDAC is highly lethal, with a five-year survival rate remaining very low despite medical advances [47]. One of the major challenges in treating PDAC is its strong resistance to chemotherapy, limiting the efficacy of conventional treatments [48–50]. Innovative targeted therapies and immunotherapies have also shown limited success, primarily due to the immunosuppressive microenvironment of PDAC [50, 51]. PDAC is characterized by a strong desmoplastic environment with a highly fibrotic stroma that expresses EDB-FN1, recognised by the L19 antibody. This makes the tumor suitable for L19-based therapies [44].

In our study, we initially screened the anti-tumor activity of L19-IL2, L19mIL12, and L19mTNF as single agents [52–54]. The first two products were effective in eradicating KPC06 tumors, while the latter was not. Interestingly, a product combining Tumor Necrosis Factor-alpha (TNFα) and IL-2 in one entity (mIL2-F8-mTNF(mut)) showed significant efficacy.

IL-2, IL-12, and TNFα are potent pro-inflammatory cytokines that have been identified as promising anti-cancer biopharmaceuticals [52, 53]. However, the efficacy of these products is often limited by their inability to preferentially localize at the disease site. The antibody-based delivery of such pro-inflammatory cytokines has emerged as a promising strategy to enhance the therapeutic index of these drugs.

L19-IL12 is currently being investigated in a Phase 1 clinical trial [NCT04471987], while mIL2-F8-mTNF(mut) is not yet available as clinical grade reagent. By contrast, L19-IL2 has been investigated in more than 300 cancer patients across multiple Phase I, II and III clinical trials [NCT01058538; NCT03705403; NCT03567889; NCT02938299; NCT05329792, NCT04362722]. Based on these considerations and on the potent anti-tumor activity observed in our initial in vivo screening, we decided to focus our work on L19-IL2 [54–57]. It is also important to note that IL-2 has emerged as a positive prognostic marker in PDAC, with evidence showing its role in amplifying the immune response and in improving patient survival [54]. IL-2 treatment may promote cell-based immunity against PDAC, not only by stimulating tumor-specific T-lymphocytes but also by enhancing dendritic cell infiltration [58–60].

Several studies have investigated the activity, as well as the mechanism of action, of IL-2 in PDAC [56, 57]. Piper et al. reported that an immunocytokine targeting Programmed Cell Death Protein-1 and fused to a variant of IL-2 (PD1-IL2v), in combination with radiotherapy (RT), improved survival rates in PDAC mouse models [61]. Our group has also reported promising antitumor activity of L19-IL2, compared to standard therapy, in xenograft orthotopic mouse models of PDAC. However, such preclinical study had to be conducted in immunocompromised mice devoid of T-lymphocytes [44]. We considered also alternative approach such as the use of PDX models reconstituted with a human immune reconstituted (HIR) system (HIR-PDX), which would allow for a more accurate representation of the human immune response. However, we anticipated challenges in obtaining a sufficient number of CD34 + cells from pancreatic cancer patients, which made it difficult to generate enough murine avatar models for all treatment groups.

Given these limitations, we have studied the effect of L19-IL2 in two syngeneic immunocompetent and orthotopic PDAC models, as the presence of T-lymphocytes is very important to properly assess the activity of immunostimulatory agents. These models are better suited to investigate the immune-modulating effects of L19-IL2 within a fully operational immune response, aligning with the central objective of our study: to enhance immune infiltration and activation within the tumor microenvironment of PDAC.

In this study, we have reported that L19-IL2 can selectively localize to and diffuse into an orthotopic PDAC lesions. This is an important achievement as pancreatic tumor cells are notoriously difficult to target, given the dense stromal component, poor vascularization, and high interstitial fluid pressures that such tumors present [58]. Moreover, L19-IL2 exhibited a clear dose-dependent anti-cancer activity. At 100 µg, L19-IL2 cured all tumor-bearing mice as single agent, while at 30 µg it only delayed tumor growth. The activity of the low dose could be however enhanced with standard chemotherapy (FOLFOX), which further reduced tumor growth and improved survival in PDAC models with low immunogenicity. Treatment with L19-IL2 led to a substantial influx of immune cells expressing activation markers such as granzymes, perforins, IL2Rb (CD122), and IL2Ra (CD25), suggesting a remodeling of a “cold” TME into a “hot” one. Notably, overexpression of GRZB and PRF1 markers induced by L19-IL2 was confirmed via multiple experimental settings, such as IF, RNA-seq, and ST.

In PDAC patients, one clinical trial evaluated L19-IL2 at 22.5 million international units (mIU) in combination with gemcitabine at 1000 mg/m^2^ but did not produce objective responses [NCT01198522]. In keeping with the results observed in this work, higher doses of L19-IL2 may be required to achieve meaningful clinical responses. Gemcitabine is now rarely used as single agent for treating PDAC patients, having been replaced by combination regimens (e.g., FOLFOX). As evidenced by the CONKO-003 study [59], FOLFOX is a more potent chemotherapy regimen that showed improved survival as a second-line treatment in patients with advanced PDAC who have experienced progression while receiving a gemcitabine-based first-line regimen.

The recommended dose of L19-IL2 in the clinic, when administered as a 2h intravenous infusion, ranges from 15 to 22.5 mIU depending on the treatment setting and on combination agents [60]. The addition of a 2h infusion of L19-IL2 may be conveniently coordinated to coincide with the 2h infusion of oxaliplatin and leucovorin in the FOLFOX regimen. It is also important to consider that the safety profile of IL-2 is distinct to that of chemotherapy. IL-2 is commonly associated with immune-related toxicities, such as capillary leak syndrome, whereas the primary toxicities of oxaliplatin and leucovorin are generally neurological, gastrointestinal, and hematological. Based on these considerations our group is planning new clinical investigations of L19-IL2 as a single agent at higher doses or, at established doses, in combination with FOLFOX.

## Conclusions

Overall, our study demonstrates that L19-IL2 selectively binds to EDB-FN1 expressed in the TME, promoting immune infiltration and activation within the tumor core and significantly enhancing the anti-tumor effects of second-line chemotherapy. These results highlight the importance of modifying the immunosuppressive microenvironment in PDAC to improve the efficacy of standard therapies. Therefore, our findings could pave the way for new clinical investigation of this innovative targeted therapy in combination with standard FOLFOX regimen.

## Supplementary Information

Supplementary Material 1: Supplementary Fig. 1 Biochemical characterization of L19-IL2. a) Schematic representation of the molecular format of L19-IL2. b) SDS Page Gel of non-covalent homodimer L19-IL2. c) Size exclusion chromatography of the dimeric product. d) In vitro L19-IL2 proliferation assay on CTTL2 cells.

Supplementary Material 2: Supplementary Fig. 2 The L19-targeted antibody specifically hits EDB-FN1 of human tumor tissues. L19-targeted antibody in IgG1 format specifically target EDB-FN1 (Green) in human PDAC tumor tissues, while KSF antibody (specific for hen egg lysozyme, an irrelevant antigen) was used as negative CTR.

Supplementary Material 3: Supplementary Fig. 3. In vivo characterization of immunocytokines sensitivity in syngeneic orthotopic mouse PDAC models. a) Plot showing tumor growth curves of KPC06 tumor-bearing mice randomly assigned to receive once a week for 2 weeks: vehicle, as CTR, L19-IL2 (100 µg/mouse), L19mIL12 (12 µg/mouse), mIL2-F8-mTNF(mut) (40 µg/mouse), L19mTNF (4 µg/mouse), standard chemotherapy with gemcitabine 10 mg/kg + abraxane 3 mg/kg (Gem/Abx). Means ± SD were reported b) Kaplan–Meier curves showing survival of KPC06 mice divided according to each experimental condition. c) Variation of body weight in the different treatment groups.

Supplementary Material 4: Supplementary Fig. 4. In vivo dose-dependent reduction of tumor volume in orthotopic mouse PDAC models upon L19-IL2 treatment. Fold reduction of tumor growth after treatment with L19-IL2 immunocytokine at high and low dose (100 µg/mouse and 30 µg/mouse, once a week for two weeks) normalized vs CTR group. Syngeneic PDAC bearing mouse models were randomly assigned to receive immunocytokines once a week for two weeks. *P*-value<0.05 was indicated in figures with one asterisk (*), *P*-value<0.01 with two asterisks (**), *P*-value<0.001 with three asterisks (***) and *P*-value<0.0001 with four asterisks (****).

Supplementary Material 5: Supplementary Fig. 5. L19-IL2 treatment effects in combination with FOLFOX in KPC12 model. a) Plot showing tumor growth curves of KPC12 tumor-bearing mice randomly assigned to receive vehicle, as CTR, standard therapy (FOLFOX i.p., once a week for 2 weeks), and L19-IL2 (30 µg/mouse i.v., once a week for 2 weeks) alone or in combination with FOLFOX. Means ± SD were reported. b) Kaplan–Meier survival analysis of KPC12 mice, grouped according to each experimental condition. c) Variation of body weight in the different treatment groups.

Supplementary Material 6: Supplementary Fig. 6. DEA showed increased immune activation of L19-IL2 compared to FOLFOX. a) Volcano plot showing the genes differentially expressed (log2 Fold Change ≦ -1.5 ≧ 1.5, FDR < 0.05) in the comparison between L19-IL2 treated and FOLFOX treated mice. b) Dot plot showing main activated and suppressed pathway in L19-IL2 treated mice (top 10 pathways). c) Network plot showing the consistent upregulation of genes involved in immune response (FDR < 0.05).

Supplementary Material 7: Supplementary Fig. 7. DEA showed increased immune activation of L19-IL2 and FOLFOX compared to FOLFOX as single agent. a) Volcano plot showing the genes differentially expressed (log2 Fold Change ≦ -1.5 ≧ 1.5, FDR < 0.05) in the comparison between Combination treated and FOLFOX treated mice. b) Dot plot showing main activated and suppressed pathways in Combination treated mice (top 10 pathways). c) Network plot showing the consistent upregulation of genes involved in immune response (FDR < 0.05).

Supplementary Material 8: Supplementary Fig. 8. Additional markers of immune activation in L19-IL2 treatment groups. Spatial clustering of Stereo-seq OMNI data on the four cores analyzed showing a major representation of immune activation markers in L19-IL2 and L19-IL + FOLFOX samples; heatmaps showing an enhanced infiltration of CD25+ CD122+ cells (a), NK cells (b), CD8+ T-lymphocytes (c), Activation markers (d) and APC cells (e).

Supplementary Material 9: Supplementary Fig. 9. Immunofluorescence analysis on KPC12 models. IF analysis for CD8+ TILs, GRZB and PRF1 in KPC12 tumor tissues. Protein analyzed (in green) and nuclei (in blue) are reported. Images shown are representative of 1 out of more than 10 fields acquired. Bar plot show percentage of positive cells grouped by treatments. *P*-value<0.05 was indicated in figures with one asterisk (*), *P*-value<0.01 with two asterisks (**), *P*-value<0.001 with three asterisks (***) and *P*-value<0.0001 with four asterisks (****).

## Data Availability

3’mRNA-Seq aggregated counts file and Stereo-Seq OMNI data are available on Zenodo. (10.5281/zenodo.12686433).

## Abbreviations

PDAC: Pancreatic Ductal Adenocarcinoma
IL-2: Interleukin-2
TERT: Telomerase reverse transcriptase
RNA-seq: RNA-sequencing
ST: Spatial transcriptomics
TME: Tumor microenvironment
ECM: Extracellular Matrix
5-FU: 5-Fluorouracil
EDB-FN1: Extra domain B of fibronectin
IF: Immunofluorescence
IHC: Immunohistochemistry
RT-PCR: Real-time PCR
TILs: Tumor-infiltrating lymphocytes
CTLs: Tumor-antigen cytotoxic T-lymphocytes
FFPE: Formalin-fixed paraffin-embedded
PFA: Paraformaldehyde
TMA: Tissue macro array
TAAs: Tumor-associated antigens
SAW: Stereo-Seq Analysis Workflow
CTR: Control
RT: Radiotherapy
GRZB: Granzyme
PRF1: Perforin
CTCF: Corrected Total Fluorescence
APCs: Antigen-presenting cells
NK: Natural killer
IL-12: Interleukin-12
TNFα: Tumor Necrosis Factor-alpha
SD: Standard Deviation
DEA: Differential Expression Analysis
GSEA: Gene Set Enrichment Analysis
