# Supplementary figures and images for: Antibody-based delivery of interleukin-2 modulates the immunosuppressive tumor microenvironment and achieves cure in pancreatic ductal adenocarcinoma syngeneic mice
Source: J Exp Clin Cancer Res. 2025 Jan 7;44:7. doi: 10.1186/s13046-024-03238-x (PMC11705946; doi:10.1186/s13046-024-03238-x)

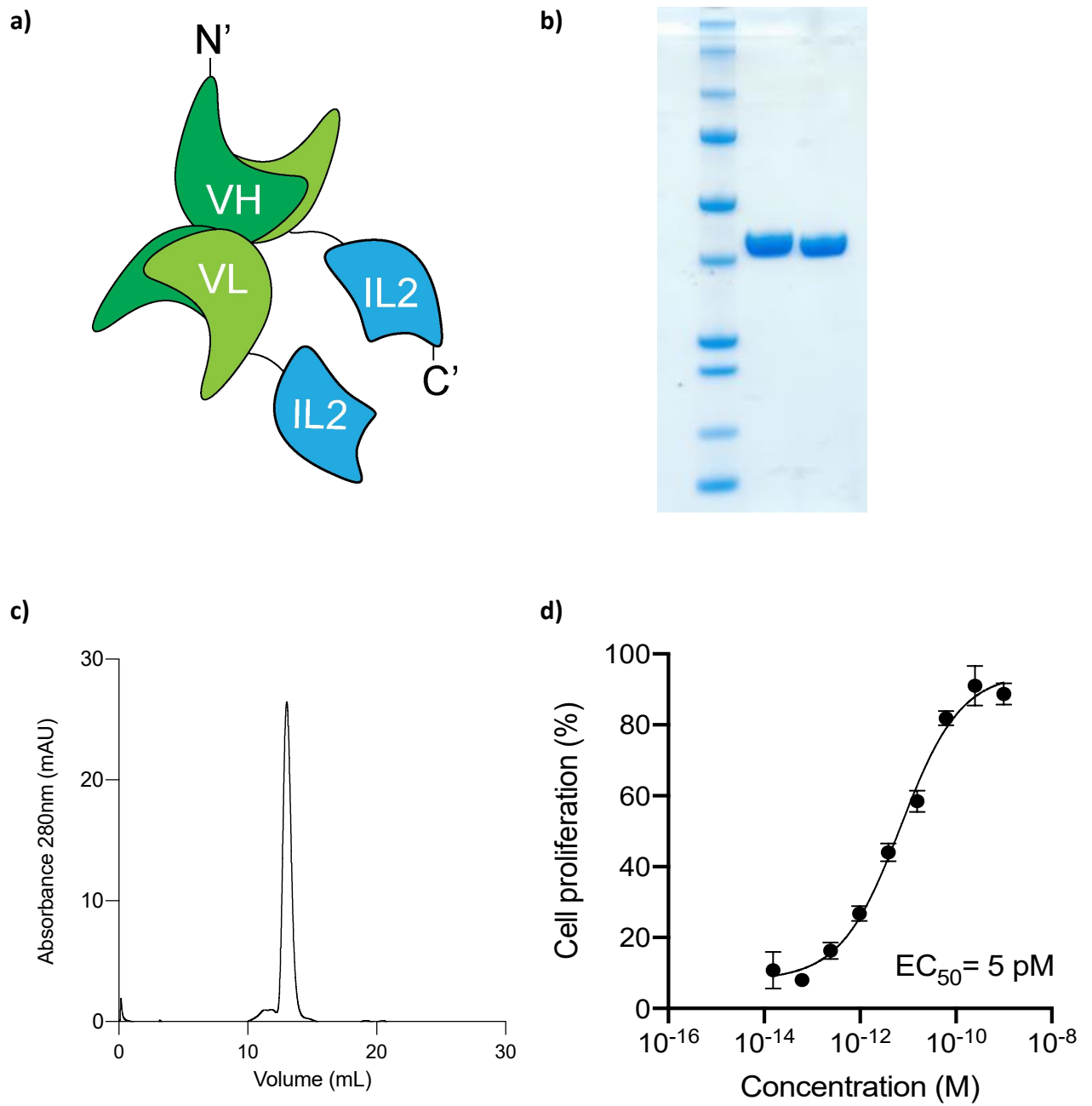

Supplement: Supplementary file 1 — Supplementary Material 1: Supplementary Fig. 1 Biochemical characterization of L19-IL2. a) Schematic representation of the molecular format of L19-IL2. b) SDS Page Gel of non-covalent homodimer L19-IL2. c) Size exclusion chromatography of the dimeric product. d) In vitro L19-IL2 proliferation assay on CTTL2 cells. [file 13046_2024_3238_MOESM1_ESM.pdf]

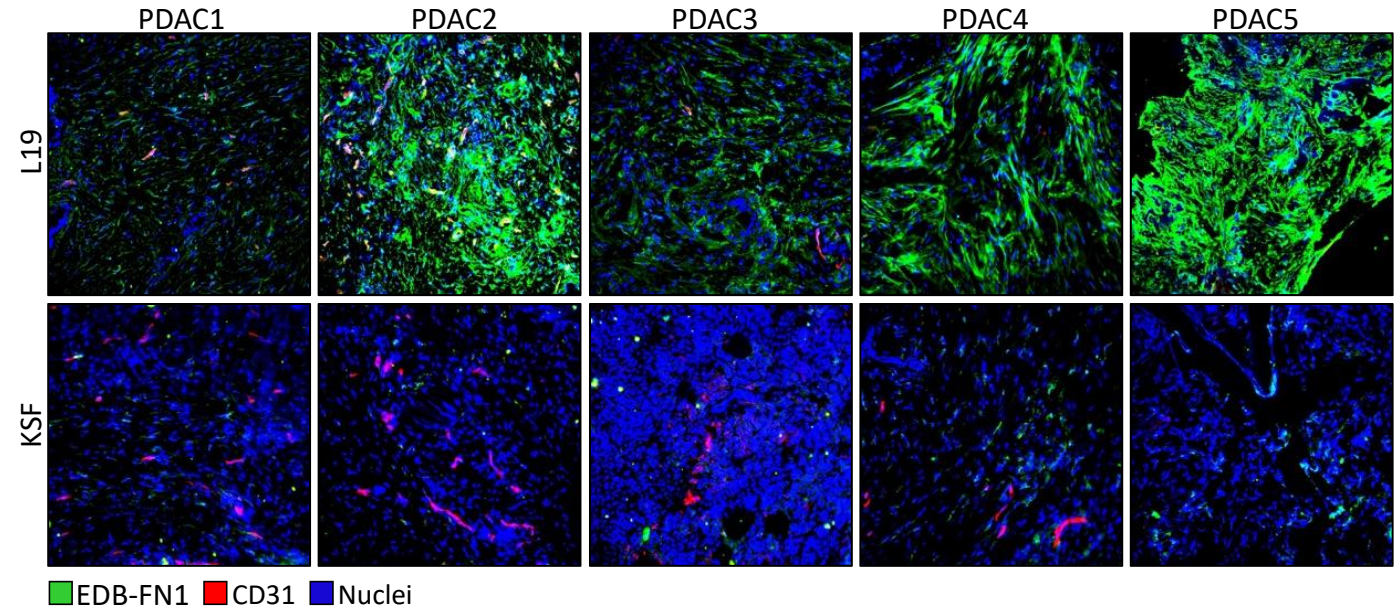

Supplement: Supplementary file 2 — Supplementary Material 2: Supplementary Fig. 2 The L19-targeted antibody specifically hits EDB-FN1 of human tumor tissues. L19-targeted antibody in IgG1 format specifically target EDB-FN1 (Green) in human PDAC tumor tissues, while KSF antibody (specific for hen egg lysozyme, an irrelevant antigen) was used as negative CTR. [file 13046_2024_3238_MOESM2_ESM.pdf]

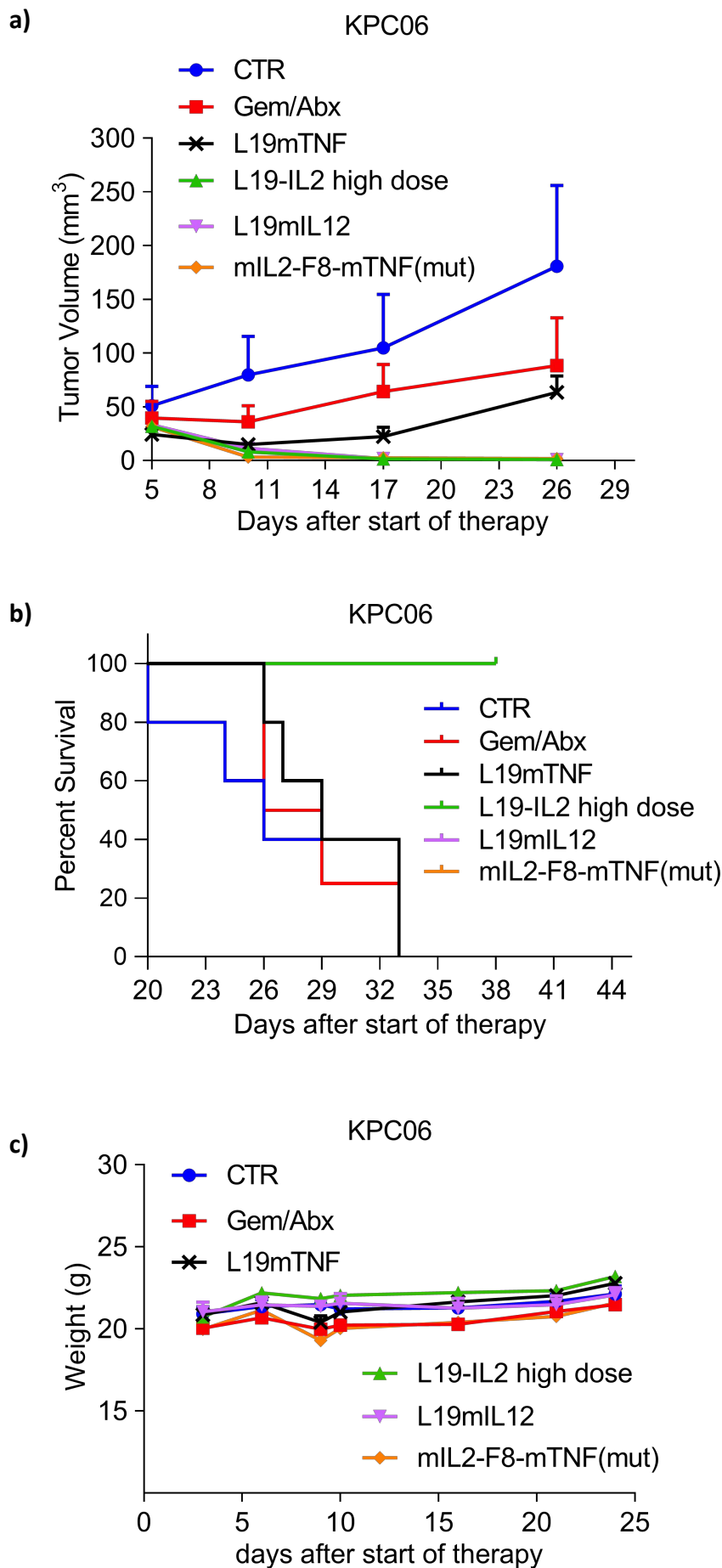

Supplement: Supplementary file 3 — Supplementary Material 3: Supplementary Fig. 3. In vivo characterization of immunocytokines sensitivity in syngeneic orthotopic mouse PDAC models. a) Plot showing tumor growth curves of KPC06 tumor-bearing mice randomly assigned to receive once a week for 2 weeks: vehicle, as CTR, L19-IL2 (100 µg/mouse), L19mIL12 (12 µg/mouse), mIL2-F8-mTNF(mut) (40 µg/mouse), L19mTNF (4 µg/mouse), standard chemotherapy with gemcitabine 10 mg/kg + abraxane 3 mg/kg (Gem/Abx). Means ± SD were reported b) Kaplan–Meier curves showing survival of KPC06 mice divided according to each experimental condition. c) Variation of body weight in the different treatment groups. [file 13046_2024_3238_MOESM3_ESM.pdf]

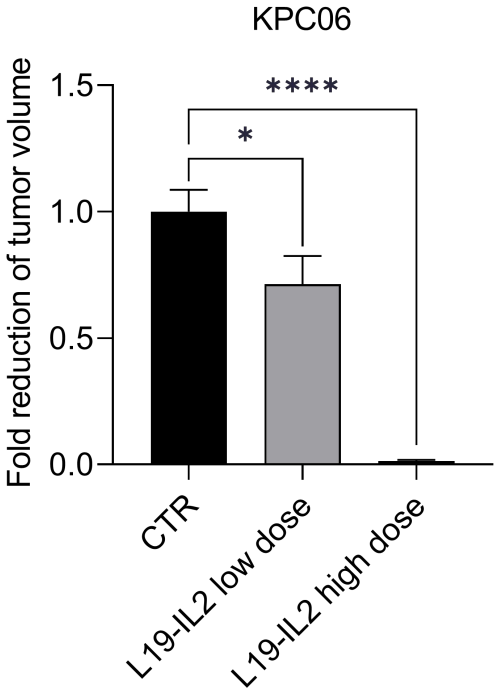

Supplement: Supplementary file 4 — Supplementary Material 4: Supplementary Fig. 4. In vivo dose-dependent reduction of tumor volume in orthotopic mouse PDAC models upon L19-IL2 treatment. Fold reduction of tumor growth after treatment with L19-IL2 immunocytokine at high and low dose (100 µg/mouse and 30 µg/mouse, once a week for two weeks) normalized vs CTR group. Syngeneic PDAC bearing mouse models were randomly assigned to receive immunocytokines once a week for two weeks. P-value<0.05 was indicated in figures with one asterisk (*), P-value<0.01 with two asterisks (**), P-value<0.001 with three asterisks (***) and P-value<0.0001 with four asterisks (****). [file 13046_2024_3238_MOESM4_ESM.pdf]

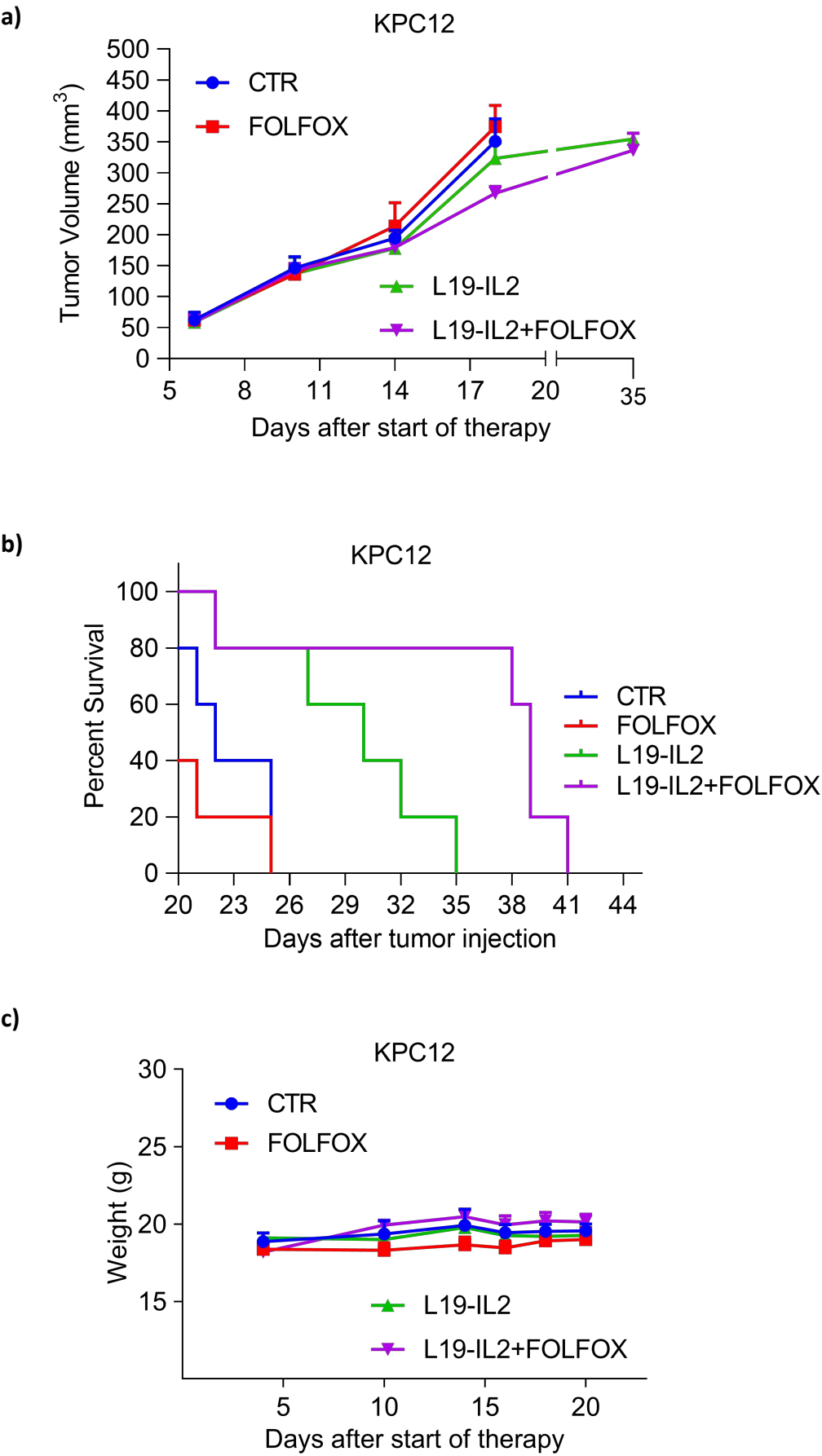

Supplement: Supplementary file 5 — Supplementary Material 5: Supplementary Fig. 5. L19-IL2 treatment effects in combination with FOLFOX in KPC12 model. a) Plot showing tumor growth curves of KPC12 tumor-bearing mice randomly assigned to receive vehicle, as CTR, standard therapy (FOLFOX i.p., once a week for 2 weeks), and L19-IL2 (30 µg/mouse i.v., once a week for 2 weeks) alone or in combination with FOLFOX. Means ± SD were reported. b) Kaplan–Meier survival analysis of KPC12 mice, grouped according to each experimental condition. c) Variation of body weight in the different treatment groups. [file 13046_2024_3238_MOESM5_ESM.pdf]

a)

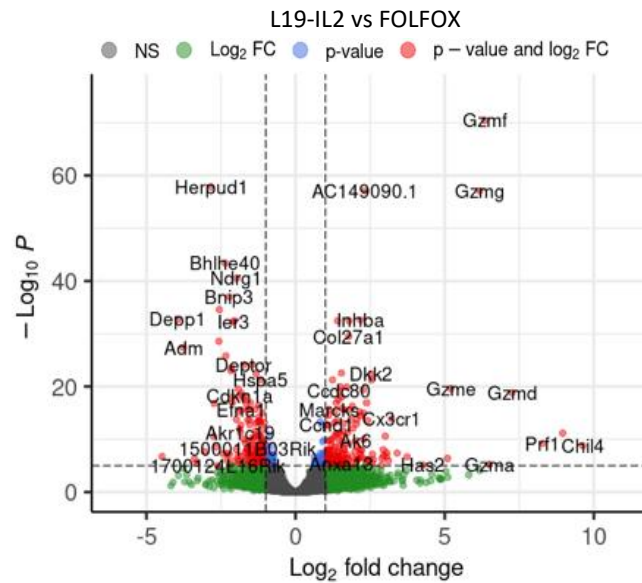

b)

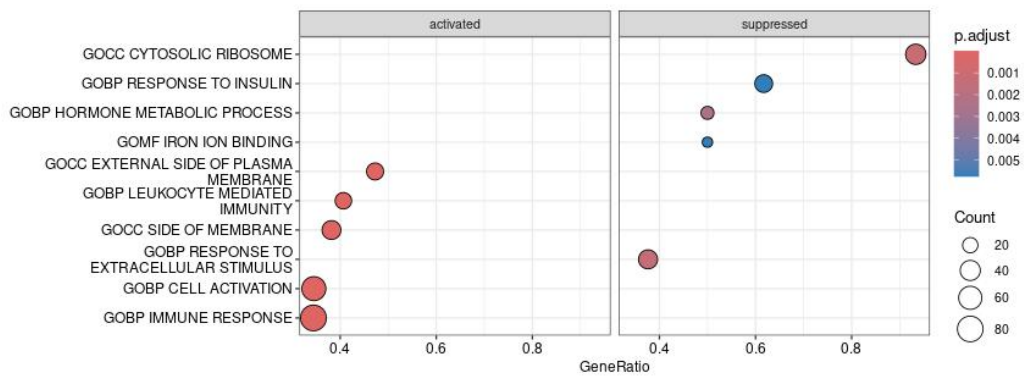

c)

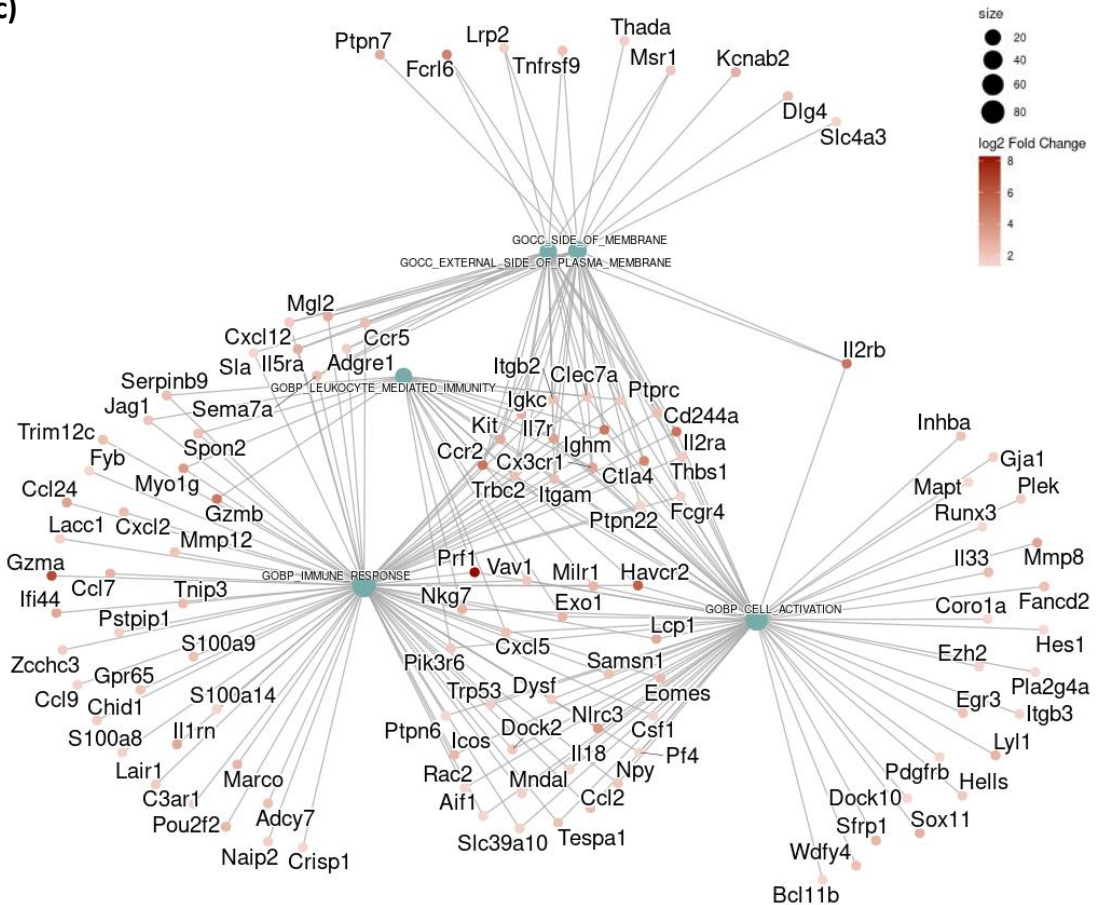

Supplement: Supplementary file 6 — Supplementary Material 6: Supplementary Fig. 6. DEA showed increased immune activation of L19-IL2 compared to FOLFOX. a) Volcano plot showing the genes differentially expressed (log2 Fold Change ≦ -1.5 ≧ 1.5, FDR < 0.05) in the comparison between L19-IL2 treated and FOLFOX treated mice. b) Dot plot showing main activated and suppressed pathway in L19-IL2 treated mice (top 10 pathways). c) Network plot showing the consistent upregulation of genes involved in immune response (FDR < 0.05). [file 13046_2024_3238_MOESM6_ESM.pdf]

a)

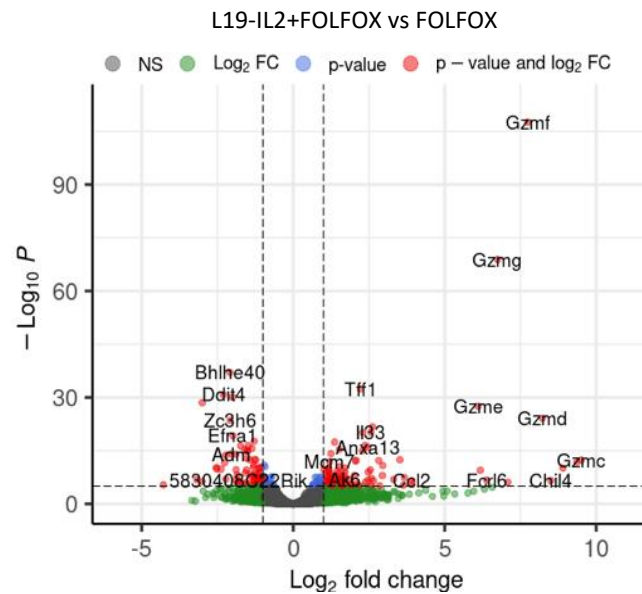

b)

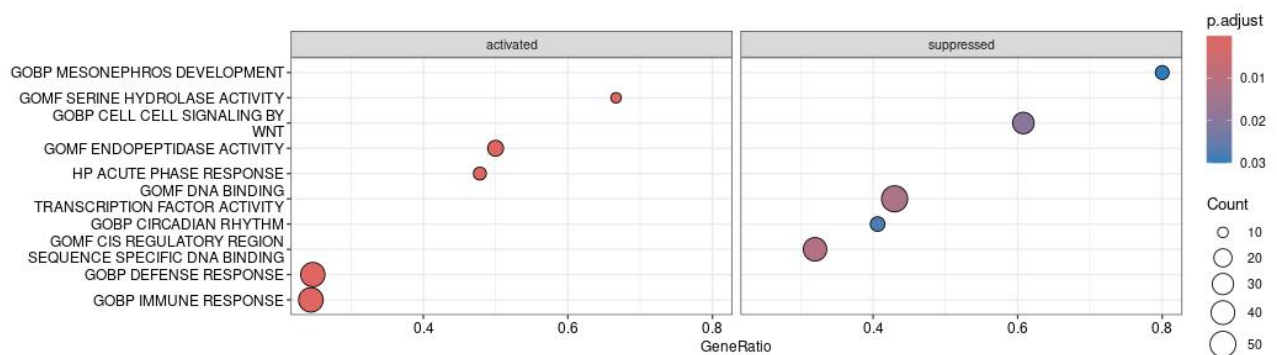

c)

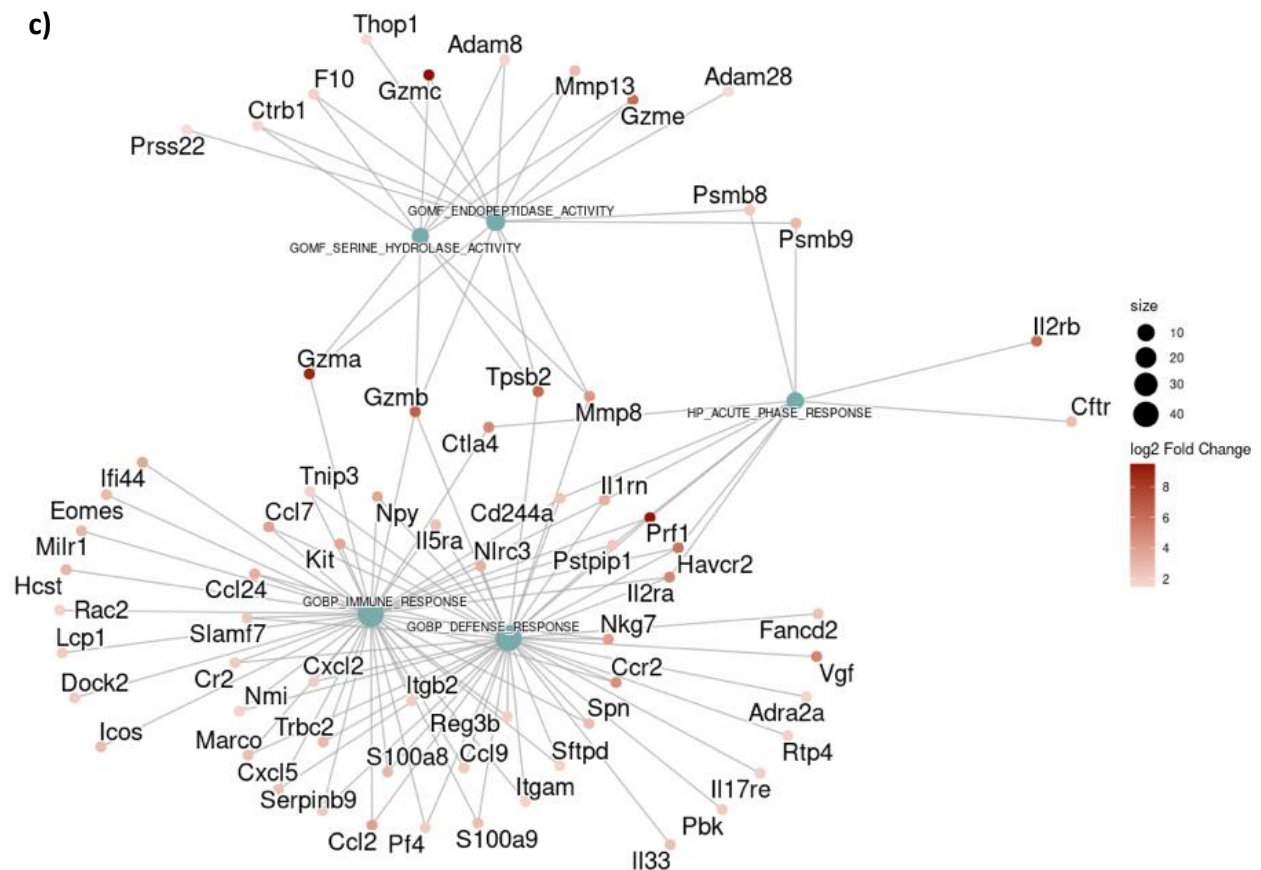

Supplement: Supplementary file 7 — Supplementary Material 7: Supplementary Fig. 7. DEA showed increased immune activation of L19-IL2 and FOLFOX compared to FOLFOX as single agent. a) Volcano plot showing the genes differentially expressed (log2 Fold Change ≦ -1.5 ≧ 1.5, FDR < 0.05) in the comparison between Combination treated and FOLFOX treated mice. b) Dot plot showing main activated and suppressed pathways in Combination treated mice (top 10 pathways). c) Network plot showing the consistent upregulation of genes involved in immune response (FDR < 0.05). [file 13046_2024_3238_MOESM7_ESM.pdf]

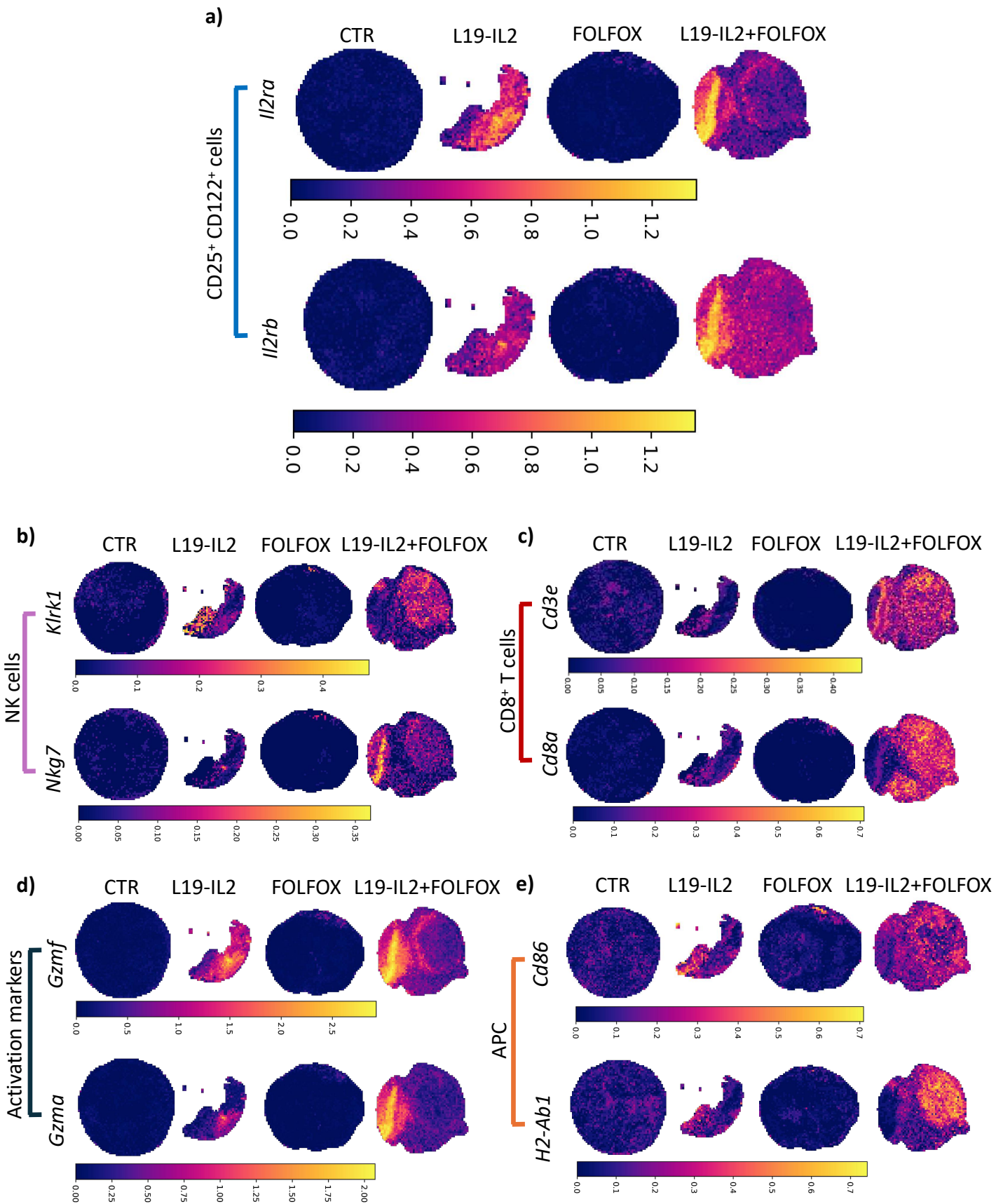

Supplement: Supplementary file 8 — Supplementary Material 8: Supplementary Fig. 8. Additional markers of immune activation in L19-IL2 treatment groups. Spatial clustering of Stereo-seq OMNI data on the four cores analyzed showing a major representation of immune activation markers in L19-IL2 and L19-IL + FOLFOX samples; heatmaps showing an enhanced infiltration of CD25+ CD122+ cells (a), NK cells (b), CD8+ T-lymphocytes (c), Activation markers (d) and APC cells (e). [file 13046_2024_3238_MOESM8_ESM.pdf]

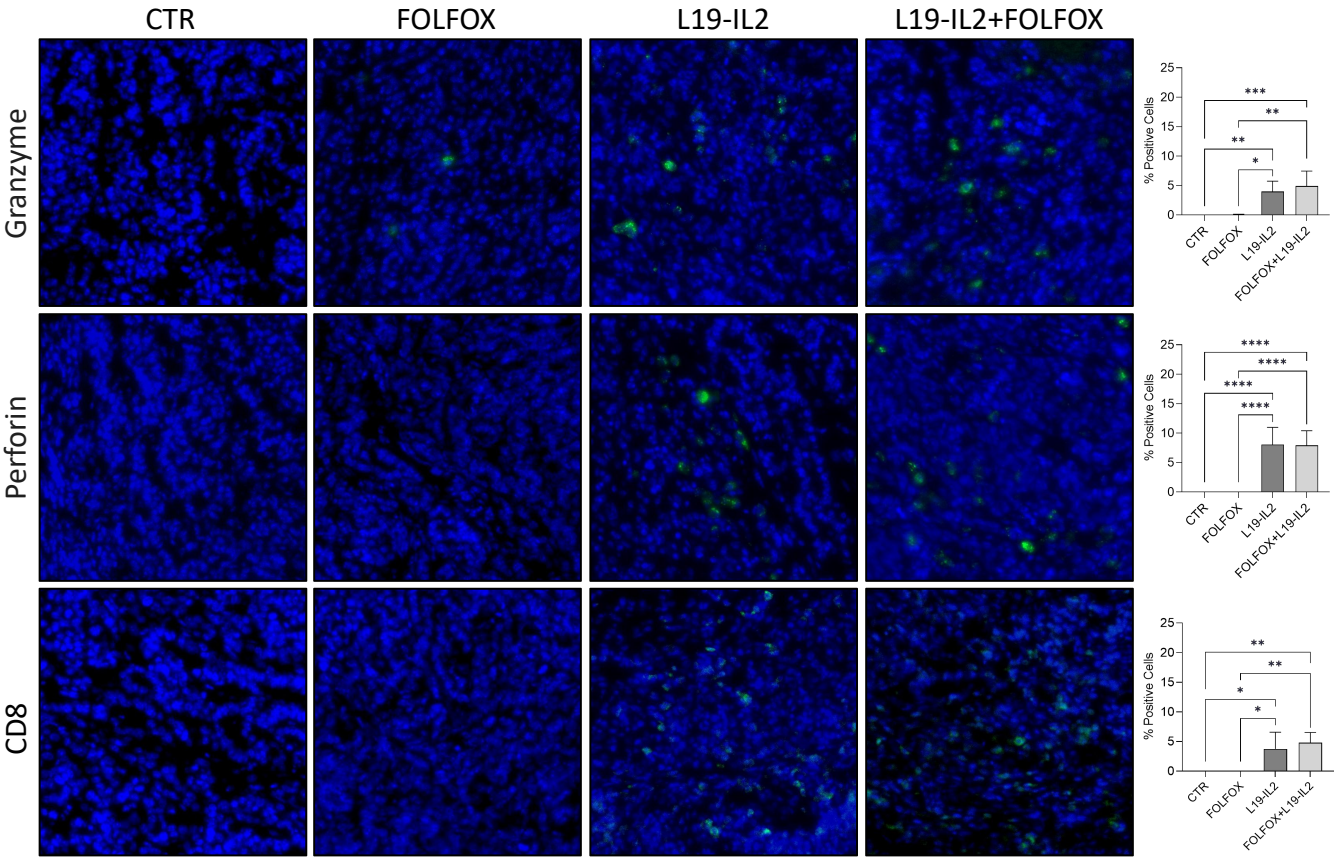

Supplement: Supplementary file 9 — Supplementary Material 9: Supplementary Fig. 9. Immunofluorescence analysis on KPC12 models. IF analysis for CD8+ TILs, GRZB and PRF1 in KPC12 tumor tissues. Protein analyzed (in green) and nuclei (in blue) are reported. Images shown are representative of 1 out of more than 10 fields acquired. Bar plot show percentage of positive cells grouped by treatments. P-value<0.05 was indicated in figures with one asterisk (*), P-value<0.01 with two asterisks (**), P-value<0.001 with three asterisks (***) and P-value<0.0001 with four asterisks (****). [file 13046_2024_3238_MOESM9_ESM.pdf]
